# Designing of a multiepitope-based vaccine against echinococcosis utilizing the potent Ag5 antigen: Immunoinformatics and simulation approaches

**DOI:** 10.1371/journal.pone.0310510

**Published:** 2025-02-12

**Authors:** Rehana Parvin, Md. Habib Ullah Masum, Homaira Pervin Heema, Aklima Akter, Mohammad Alamgir Hossain, A. M. A. M. Zonaed Siddiki

**Affiliations:** 1 Genomics Research Group, Department of Pathology and Parasitology, Faculty of Veterinary Medicine, Chattogram Veterinary and Animal Sciences University (CVASU), Chattogram, Bangladesh; 2 Department of Genomics and Bioinformatics, Faculty of Biotechnology and Genetic Engineering, Chattogram Veterinary and Animal Sciences University (CVASU), Chattogram, Bangladesh; 3 Nextgen Informatics Ltd, Bangladesh; Government College University Faisalabad, PAKISTAN

## Abstract

Echinococcosis is a significant parasitic zoonotic disease with severe implications for human and animal health. To date, there has been no effective vaccine candidate available for echinococcosis. Therefore, we employed computational approaches to develop a multiepitope-based vaccine using the most potent epitopes of MHC-I, MHC-II, and B-cell derived from the Ag5 protein of *Echinococcus* spp. The final vaccine construct containing the epitopes, linkers, and adjuvant exhibited potent antigenicity (score > 0.1) with no evidence of allergenicity (score < 0) and toxicity (score < 0) in several computational platforms. The vaccine also exhibited favorable physicochemical characteristics such as being highly soluble (SOLpro score of 0.781243) and hydrophilic (Grand average of hydropathy of -0.433). Moreover, the tertiary structure of the vaccine was also found to be structurally stable, with a Z score of -5.71. Further, the molecular docking analysis confirmed the vaccine’s significant binding affinity to the RP-105 (docking score of -1252.7) and TLR-9 (docking score of -970.9). The molecular dynamic simulations confirmed the structural stability of the docked complexes under a virtual physiological system. The negative ΔTOTAL values derived from the MM-PBSA and MM-GBSA analyses confirmed a spontaneous and thermodynamically favorable binding process between the vaccine and receptors. Moreover, the vaccine demonstrated high potentiality to elicit both innate (natural killer cell, dendritic and macrophage) and adaptive (B-cell, helper T cell and cytotoxic T cell) immune responses with sustained humoral immune responses evidenced by increased IFN-γ and IL-2 levels. Following codon optimization and *in silico* cloning, the vaccine was successfully expressed (CAI value of 0.9607 and average GC content of 52.34%) after being inserted into the pET-28a (+) plasmid of *E*. *coli*. These findings highlight the potential of the designed vaccine candidate to combat echinococcosis and lay the groundwork for future preclinical and clinical studies.

## 1. Introduction

Hydatid cystic disease, or echinococcosis, is one of the most significant emerging zoonotic tapeworm diseases globally [1]. This disease is mainly manifested by a meta-cestode tapeworm known as *Echinococcus* spp. [2]. However, this disease also has socioeconomic impacts in different endemic areas, especially on humans and many other herbivores [3]. In Bangladesh, echinococcosis has been reported extensively [3, 4], and the government has classified it as a significant parasitic zoonosis, 13th among all zoonoses in this territory [5]. The hydatid cysts of *Echinococcus* spp. are the pathogenic forms that infect the liver hepatocytes and multiple organs of cattle, sheep, and goats. Active transmission has been reported in humans with clinical features including hepatic and abdominal dysfunction [6]. The parasitic infection contributes to significant economic losses in the livestock industry due to its high treatment costs, loss of production, condemnation of internal organs, and death of the infected animals [7, 8]. According to the most recent taxonomic update, there are nine identified species of *Echinococcus* spp. and two predominant forms of the illness, including cystic echinococcosis (CE) and alveolar echinococcosis (AE) [9]. The CE is mainly manifested by *Echinococcus granulosus*, whereas *Echinococcus multilocularis* primarily causes AE. However, both the CE and AE have potent zoonotic features and are generally transmitted by dogs and foxes [10, 11]. The life cycle of this parasite requires two hosts following three developmental stages. The definitive hosts are carnivores, especially dogs [12], while the intermediate hosts are omnivores such as sheep, cattle, goats, buffalo, pigs, camels, deer, horses, and humans [4]. Contaminated foods and water can lead to human infection through the ingestion of the parasite’s eggs [13]. As aforementioned, *Echinococcus* spp. mainly infects the hepatocyte of the liver, but it can also produce systemic diseases in different organs, including the lungs, abdominal cavity, brain, heart, bone, and kidney [6, 14]. However, when this disease is left untreated, it can lead to a life-threatening condition [8]. The early diagnosis of this disease is crucial due to the small parasitic cyst and is challenging to confirm, even with imaging. Current treatment of this disease primarily relies on chemotherapy for early-infected patients, while surgical interventions are required for late-stage patients. These limited treatment options are the main reasons for the high incidence of the disease. The use of anthelmintic drugs and pharmacotherapy is not always effective and can lead to unavoidable side effects [15]. Due to its highly zoonotic nature and complex life cycle, it is difficult to interrupt the transmission of this disease from animal to human. Developing new drugs and vaccines is essential for effectively addressing and controlling the echinococcosis [16]. A protein called antigen 5 (Ag5) has been observed in the parasite’s larval and adult stages, and it could be a vital tool for developing an effective vaccine against this disease [15].

Designing novel vaccines consisting of multiple epitopes has become a new approach to elicit both humoral and cellular immunity to enhance the host’s protective immune responses [17, 18]. Immunoinformatics is crucial in developing multiepitope vaccines against infectious diseases and their causative agents, including bacteria, viruses, and parasites [19–25]. It utilizes computational algorithms and databases to identify immunogenic epitopes, predict antigen processing and presentation, and evaluate vaccine effectiveness and safety. This multidisciplinary approach facilitates epitope selection, antigen construction, and immunogenicity prediction, leading to the development of vaccines with enhanced efficacy, broad-spectrum protection, and reduced risk of immune evasion. By integrating genomics, proteomics, and immunology data, immunoinformatics enables the rational design of vaccines to elicit robust cellular and humoral immune responses against diverse pathogens [26–29]. Several multiepitope vaccine candidates have been designed for echinococcosis targeting multiple proteins, including EgTeg, EgFABP1, EmEMY162, EmLAP, EmGLUT1, Em-TSP3 EgA31, and EgG1Y162. These vaccines showed potent immune responses in different experimental trials [16, 30–32]. However, neither did these vaccines target the dominant *Echinococcus* spp, *E*. *granulosus*, and *E*. *multilocularis*, nor did they target both diseases. The Ag5 protein, on the other hand, is a thermo-labile complex glycoprotein with a concanavalin A binding structure and has been recognized as a dominant component of the *E*. *granulosus* and *E*. *multilocularis* cyst fluid [15]. It is highly expressed in the protoscolex tegument, the egg’s embryonic membrane and the oncosphere’s surface of the parasite [9]. As the protein is expressed in all stages of the parasite’s life cycle, it has been identified as a highly immunogenic antigen in human infections. These unique characteristics make it an ideal candidate for vaccine design, not only for the intermediate but also for the definitive hosts [15].

This study aims to design a chimeric multiepitope-based vaccine against echinococcosis made up of several epitopes using reverse vaccinology approaches. Therefore, we predicted MHC-I (Major histocompatibility complex-I), MHC-II (Major histocompatibility complex-II), and B-cell binding epitopes from the Ag5 proteins using next-generation reverse vaccinology approaches. This study will establish a foundation for developing vaccines based on multiple epitopes that fight echinococcosis in cattle and halt the zoonotic feature of the disease.

## 2. Method

### 2.1 Sequence retrieval

The amino acid sequences of the Ag5 proteins of *E*. *granulosus* (Accession no. CDS21435.1) and *E*. *multilocularis* (Accession no. CDI98027.1) were retrieved from National Center for Biotechnology Information (NCBI) (https://www.ncbi.nlm.nih.gov/) protein database and stored in FASTA format (Information and database, 2021) to develop a multi-epitope vaccine against echinococcosis.

### 2.2 Pairwise sequence alignment

The pairwise sequence alignment between the Ag5 proteins of the selected isolates was performed by the EMBL-EBI server [33]. The matchen EMBOSS tools of the EMBL-EBI were utilized for further pairwise sequence similarity search [34]. The conserved regions of the aligned sequences were then applied for subsequent MHC-I, MHC-II, and liner B-cell predictions [35, 36].

### 2.3 MHC-I binding epitope prediction

The selected conserved region of the Ag5 proteins was used to predict all possible MHC-I binding epitopes by the Immune Epitope Database (IEDB) [35–37]. The server contains information on antibodies, B-cell and T-cell epitopes, MHC epitopes, and MHC binding ligands. The conserved FASTA sequence of the targeted protein (Ag5) was uploaded to the servers during the prediction. 105 bovine lymphocyte antigens (BoLA) alleles (Cattle alleles) were chosen while predicting the MHC-I binding epitopes. The predicted epitopes were further evaluated for antigenicity, allergenicity, and toxicity. The antigenicity was predicted by the VaxiJen v2.0 server [38], while the AllerTop v.2.0 (https://www.ddg-pharmfac.net/AllerTOP/) [39], AllergenFP (https://ddg-pharmfac.net/AllergenFP/) [40] and Allermatch (https://www.allermatch.org/) [41] server were utilized for the allergenicity prediction. The epitopes recognized as probable antigens and non-allergens were applied for toxicity prediction by the ToxinPred (http://crdd.osdd.net/raghava/toxinpred/) server [42, 43]. Only non-toxic epitopes were selected for subsequent vaccine development by discarding all possible toxic epitopes.

### 2.4 MHC-II binding epitope prediction

The MHC-II binding epitopes were predicted by using the conserved region of the Ag5 proteins through the IEDB [44] and NetMHCIIpan 4.0 server (http://www.cbs.dtu.dk/services/NetMHCIIpan/) [36]. During this prediction, eight human leukocyte antigen–DR isotype (HLA-DR) alleles were selected, including HLA-DRB1*0301, HLA-DRB1*0401, HLA-DRB1*0801, HLA-DRB1*1101, HLA-DRB1*1301, HLADRB1*1401, HLA-DRB3*0101, and HLA-DRB3*0201 [45]. To date, no cattle-specific MHC-II alleles are available in any allele database. As a result, the human alleles were chosen due to their pseudo-sequence similarity to the BoLA alleles [45]. During this prediction, eight human HLA-DR alleles were selected to predict MHC-II binding epitopes. The predicted epitopes were also assessed for antigenicity, allergenicity, and toxicity prediction. The VaxiJen v2.0 [38] were utilized for antigenicity prediction, while AllerTop v.2.0 (https://www.ddg-pharmfac.net/AllerTOP/) [39], AllergenFP [40] and Allermatch [41], were utilized for the allergenicity prediction. Subsequently, the toxicity of the selected epitopes was predicted by the ToxinPred server [42, 43].

### 2.5 B-cell epitopes prediction

Identifying a linear sequence comprising B-cell epitopes within a protein sequence is a prerequisite for epitope prediction. The linear B-cell epitopes of the targeted proteins (Ag5) were predicted by the IEDB and the Bepipred 2.0 server [46]. The IEDB server uses a predictive strategy to recognize linear B-cell epitopes by analyzing the sequence characteristics of the antigen via amino acid scales and emini surface accessibility [47]. Moreover, the BepiPred server uses a hidden Markov model (HMM) and propensity scale method [46]. Afterwards, the antigenicity of the selected epitopes was predicted by using VaxiJen v2.0 [38]. Subsequently, the AllerTop v.2.0 [39] and AllergenFP were utilized for the allergenicity prediction [40]. The toxicity of the selected epitopes was further predicted by the ToxinPred server [42, 43].

### 2.6 Mapping the vaccine construct

Only the most favorable epitopes from Ag5 proteins were rigorously chosen in the final vaccine design. The primary objective of the vaccine design was to add appropriate adjuvants to the selected epitopes. Two adjuvants, bovine beta-defensin for vaccine 1 (V1) and heparin-binding hemagglutinin (HBHA) for vaccine 2 (V2) were utilized for the final vaccine construction. The linkers EAAAK, AYY, AK, and GPGPG were used to integrate the selected epitopes with the adjuvants [48].

### 2.7 Evaluation of physicochemical properties, solubility, allergenicity, and antigenicity

The physicochemical characteristics of the V1 and V2 were predicted using Expasy’s ProtParam online server (http://web.expasy.org/protparam/) [49]. The properties encompassed a combination of positive and negative residues, total amino acids, atom number and composition, molecular weight, molecular formula, instability index, aliphatic index, isoelectric point (pI), extinction coefficients, and overall hydropathicity (GRAVY). The solubility of V1 and V2 was assessed using SOLpro (http://scratch.proteomics.ics.uci.edu) [50]. Also, the potential allergenicity of the V1 and V2 was assessed by using AllergenFP [40], AllerTOP v. 2.0 [39] and AlgPred [51] server. The antigenicity of the vaccines was also validated by the SCRATCH server [52] and VaxiJen v2.0 [38].

### 2.8 Secondary structure prediction of the vaccine constructs

The secondary structure of the vaccines was predicted by the SOPMA, GOR4 and PSIPRED servers [53–55]. The SOPMA and GOR4 servers predict the amino acids in a protein sequence with a 69.5% accuracy and provide a three-state depiction of the secondary structure, including alpha-helix, beta-sheet, and coil regions [53]. Furthermore, the PSIPRED server evaluates the results of protein-specific iterated BLAST (PSI-BLAST) using two feed-forward neural networks [56].

### 2.9 Tertiary structure prediction of the vaccine constructs, refinement, and validation

The tertiary structure (3D) of the V1 and V2 were predicted by the I-TASSER server [57]. The server generates a precise tertiary structure using multiple threading alignments and repeated template fragment assembly simulations [57]. The confidence score (C-score) has been considered while determining the quality of any predicted 3D structure. A higher C-score indicates more confidence in the accuracy of a model’s predictions. Furthermore, the TM-score and root mean square deviation (RMSD) values suggest higher resolution and improved alignment of the projected 3D model [57, 58]. GalaxyWEB subsequently utilized the vaccine’s 3D model for structural refinement [59]. The validation of the predicted refined structures was confirmed by the SAVES (https://saves.mbi.ucla.edu/) server by utilizing the algorithm including PROCHECK [60], ERRAT [61], and VERIFY 3D [62, 63]. Potential errors in the predicted 3D model structures were also screened using the ProSA-web server (https://prosa.services.came.sbg.ac.at/prosa.php) [64]. The server-calculated Z-score characterizes how precise the model framework is [65]. Following these assessments, only the V2 was chosen for further analysis.

### 2.10 Prediction of discontinuous B-cell epitopes

Utilizing Ellipro of the IEDB database (http://tools.iedb.org/ellipro/), discontinuous B-cell epitopes were predicted using the 3D structure of the V2. The server applies three algorithms while predicting the discontinuous B-cell epitopes and provides protrusion index (PI) values to compute the protein shape, the residue PI quantification, and adjacent cluster residues [66].

### 2.11 Molecular docking of the vaccine constructs

Before the protein-protein docking analysis (www.rcsb.org), the 3D of the bovine radioprotective 105 (RP-105) and toll-like receptor 9 (TLR-9) were obtained from the PDB database with the codes of 3RG1 and 5Y3M, respectively. The RP-105 receptor is crucial in vaccine-mediated immune response, particularly in stimulating and activating macrophages, dendritic cells, and B-cells to combat pathogenic microbes. Thus, it activates innate and adaptive immune responses [67–70]. Meanwhile, the TLR-9 interacts with pathogen-associated molecular patterns (PAPMs) or microbial peptides to produce cytokine responses, which regulate innate and adaptive immune responses [71]. The protein-protein docking analyses associated with the V2 and the receptors (TLR-9 and RP-105) were then performed using ClusPro 2.0 server [72–74]. The server uses RMSD-based clustering, energy minimization-based refining, and rigid body docking while performing protein-protein docking analysis [72–74].

### 2.12 Molecular dynamic (MD) simulation

The MD simulation was run to minimize and evaluate the stability of the V2-RP-105 and V2-TLR-9 complexes within a virtually designed physiological environment. Using the assistant model building with energy refinement (AMBER 18) [75], and ff19SB force field [76] with OPC water model [77, 78], the MD simulation was performed. Octahedron box shape was utilized with the V2, V2-RP-105, and V2-TLR-9 complexes at least 12Å away from the edge of the water-filled box to achieve a three-layer solvation [79]. Using Amber’s "tleap" package, Na+ and Cl− counter ions were added to neutralize the system. The systems were minimized at 500 cycles of the steepest descent and 1000 steps of a conjugate gradient to eliminate any constraint atom. To achieve conformational stability, the systems were heated for 50 picoseconds (ps) using langevin dynamics to maintain a constant temperature of 300 K. They were then equilibrated for 5 nanoseconds (ns) at temperature and pressure with isotropic position scaling. Using the SHAKE and particle-mesh ewald (PME) methods, all simulation was run for 100ns in pmemd.cuda [80, 81]. For long-term interactions, a non-bond contacts cut-off radius of 10Å was applied.

### 2.13 Free energy calculation by molecular mechanics poisson-boltzmann surface area (MM-PBSA) and molecular mechanics with generalized born and surface area solvation (MM-GBSA) approaches

The binding free energies of the V2-RP-105 and V2-TLR-9 complexes were calculated in terms of MM-PBSA and MM-GBSA. Here, the MMPBSA.py package of AMBER 18 program was used to run the MMPBSA model [82], while the MM-GBSA model was executed by the HawkDock tool [83–86]. Both cases include the evaluation of several molecular mechanics approaches, including the study of binding contacts (ΔTOTAL), electrostatic interactions (ΔEEL), van der Waals forces (ΔVDWAALS), polar (ΔEGB) and non-polar (ΔESURF) components [83–85].

### 2.14 Normal mode analysis (NMA) of the vaccine-receptor complexes

The NMA describes the collective functional motions of macromolecules [17, 87, 88]. Therefore, the iMODS server was applied to run NMA of the V2- RP-105 and V2-TLR-9 complexes [88]. The server provides multiple potent motion configurations, such as affine-model arrows, vector fields, and modal animations. The server computes various properties, including mobility (B-factor), deformability, eigenvalues, covariance map, and linkage matrix [88]. The B-factor explains how atoms are disrupted from their structural equilibrium. The deformability plot is a graphical depiction of protein flexibility, especially coil or domain linkers. Furthermore, the eigenvalue provides a measure of the complex’s stability, with higher values indicating an increased level of stability [87, 88].

### 2.15 Codon optimization and *in-silico* cloning

Codon optimization is essential while assessing the genetic code’s degeneracy [89]. The *E*. *coli* strain K12 codon system was subsequently implemented using the Java Codon Adaptation Tool (JCat) server (http://www.jcat.de/Start.jsp). The server uses the codon adaptation index (CAI) and GC content. A CAI of 0.8 or greater is exceptional, as GC levels may range from 30% to 70% [89, 90]. The *E*. *coli* plasmid vector pET-28a (+) was used to clone the optimized multi-epitope vaccine gene sequence. EcoRI and BgII restriction sites were then ligated to the N and C terminals of the sequence, respectively. After restriction site ligation, The SnapGene program was used to insert the optimized sequence of the final vaccine design into the plasmid vector pET-28a (+) for evaluating vaccine expression.

### 2.16 Immune simulation

The C-ImmSim server was utilized to run the immune simulation of the vaccine [91]. C-ImmSim may provide insight into the way a mammalian immune system responds to a proposed vaccine at the humoral (antibody-mediated) and cellular (cell-mediated) levels [91, 92]. For administration, three doses of the multi-epitope vaccine were chosen, and each dosage was given at intervals of four weeks. The number of adjuvants and antigen injections was set at 100 and 1000, respectively, and the simulation parameters remained at default settings. The time intervals were set at 1, 28, and 56 days. In addition, the simulation’s settings were modified, with the number of steps being 1000 and the volume being set to 50. It is significant to highlight that lipopolysaccharides (LPS) were not included in the immunization injection and that 12345 was the random seed utilized in this investigation.

## 3. Results

### 3.1 Amino acids sequence retrieval and pairwise and local alignment

**Fig 1:**
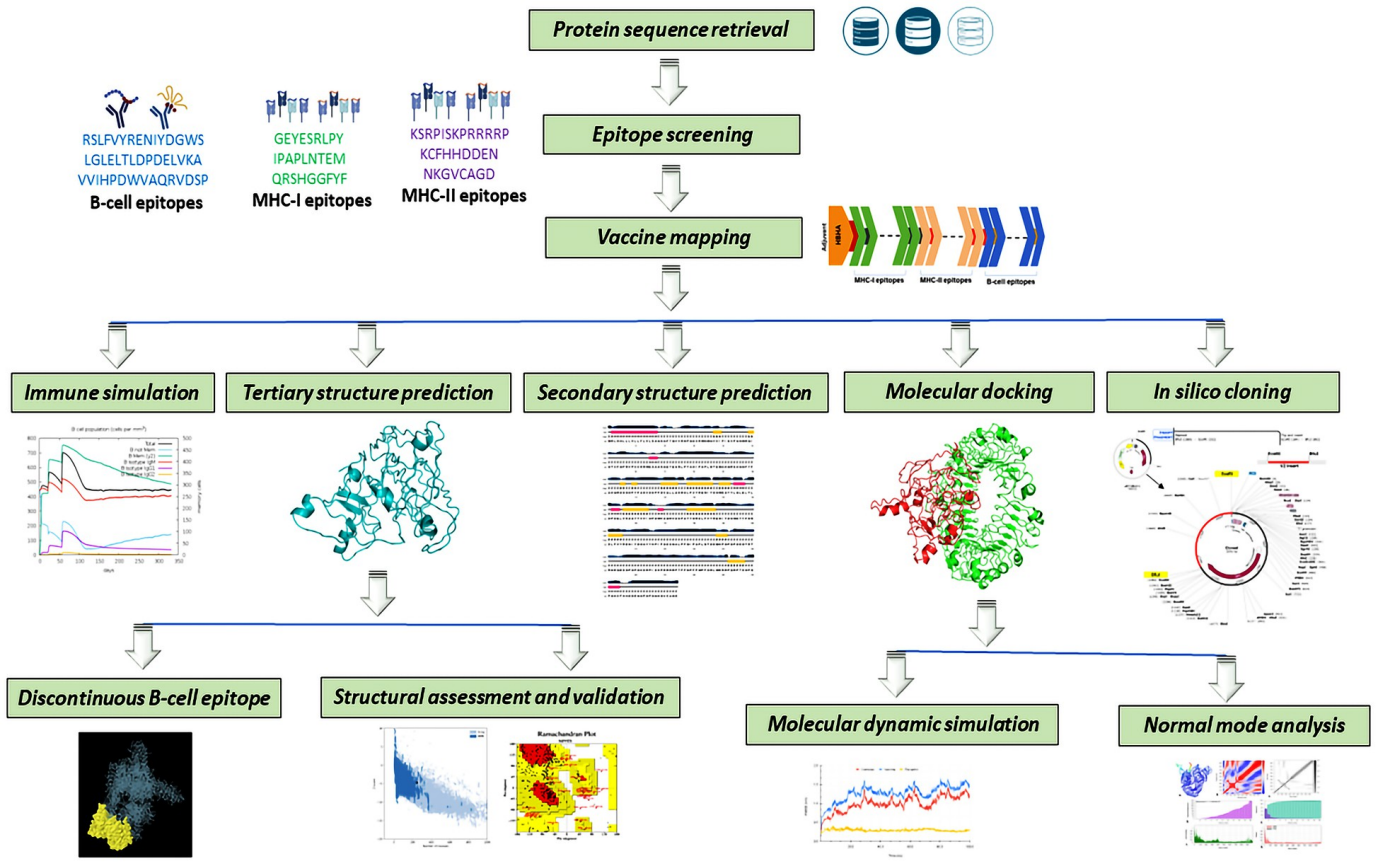
The overview of the multiepitope-based vaccine design for echinococcosis.

At first, the pairwise sequence alignment of the retrieved amino acid sequences of the Ag5 proteins (*E*. *granulosus* and *E*. *multilocularis*) was performed by the EMBL-EBI alignment tool. The EMBL-EBI gathered these retrieved sequences for pairwise sequence alignment, and the Matchen EMBOSS executed the local alignment. Subsequently, the length of the amino acid sequence (484), identity (96.7%), similarity (98.1%), and score (2550) were identified (S1 Fig). A summary of the work is depicted in Fig 1.

### 3.2 MHC-I binding epitopes prediction

Using the Ag5 protein peptide antigens, the IEDB server MHC-I prediction program calculated every potential MHC-I binding epitope for the applied proteins. Based on their percentile rank and affinity score, five peptides, each with a length of ten mer, were chosen for vaccine development (Table 1). 105 BoLA alleles were chosen to predict MHC class I binding epitopes. The epitopes were also passed on other parameters, including allergenicity, antigenicity, immunogenicity, and toxicity, where these are found to be probable non-allergen, probable antigen, probable immunogen, and non-toxic. Table 1 displays the findings, which include a list of the top five high-scoring epitopes.

**Table 1:** List of selected epitopes of the Ag5 with their antigenicity, allergenicity, and toxicity assessment of the five best MHC- I epitopes.

Allele	Peptide	Start	End	Percen-tile rank	Allergenicity	Antigenicity (Vaxigen score)	Immuno-genecity	Toxicity
BoLA-T5	GEYESRLPY	176	185	0.19	Probable non-allergen	0.5765	0.06883	Non-toxin
BoLA-3:01001	IPAPLNTEM	259	268	0.2	Probable non-allergen	0.4718	0.0982	Non-toxin
BoLA-2:04801	QRSHGGFYF	35	44	0.24	Probable non-allergen	0.3912	0.05907	Non-toxin
BoLA-2:02201	QRSHGGFYF	35	44	0.29	Probable non-allergen	0.3912	0.05907	Non-toxin
BoLA-JSP.1	VDSPFDVALL	311	320	0.36	Probable non-allergen	1.1342	0.13614	Non-toxin

### 3.3 MHC-II binding epitopes prediction

A total number of five peptides, each with a length of fifteen mer, were predicted utilizing the IEDB server’s MHC-II prediction program with the parameters of eight human HLA-DR alleles including HLA-DRB1*0301, HLA-DRB1*0401, HLA-DRB1*0801, HLA-DRB1*1101, HLA-DRB1*1301, HLADRB1*1401, HLA-DRB3*0101 and HLA-DRB3*0201. The selected epitopes were also passed parameters such as allergenicity, antigenicity, and toxicity while conferring probable non-allergen, probable antigen, and non-toxic substance (Table 2). These peptide sequences were subsequently utilized for constructing epitope vaccines.

**Table 2:** List of selected epitopes of the Ag5 with their antigenicity, allergenicity, and toxicity assessment of the five best MHC -II epitopes.

Peptide	Start	End	Percentile rank	Allergenicity	Antigenicity (Vaxigen score)	Toxicity
RSLFVYRENIYDGWS	56	70	0.28	Probable non-allergen	0.1704	Non-toxin
LGLELTLDPDELVKA	20	34	0.34	Probable non-allergen	0.3343	Non-toxin
VVIHPDWVAQRVDSP	300	314	0.35	Probable non-allergen	0.3200	Non-toxin
NRSLFVYRENIYDGW	55	69	0.37	Probable non-allergen	0.4893	Non-toxin
FNRSLFVYRENIYDG	54	68	0.37	Probable non-allergen	0.8934	Non-toxin

### 3.4 Linear B-cell epitope prediction

Using the IEDB and Bepipred 2.0, five peptide sequences of the Ag5 protein were selected as linear B-cell epitopes (Table 3). The three servers, AllerTOP, AllergenFP, and Allermatch, could anticipate which epitopes might trigger allergic reactions. Epitopes identified as non-allergens by a minimum of two servers were chosen. The toxicity and antigenicity of the selected epitopes were assessed using ToxinPred and VaxiJen 2.0 servers, respectively. The epitopes predicted as toxic were also removed.

**Table 3:** Selected linear B cell epitopes and their allergenicity and antigenicity.

Peptide	Peptide length	Antigenicity (Vaxijen score)	Allergenicity	Toxicity
KPIFGSSNALPFGIPAPLNTDEMKP	25	0.5450	Probable non-allergen	Non-toxin
EGTRTRNEQES	11	1.2980	Probable Allergen	Non-toxin
KSRPISKPRRRRPTFFNPFFWPFGRLWERRPQRPTS	36	1.0212	Probable non-allergen	Non-toxin
KCFHHDDEN	9	0.6257	Probable non-allergen	Non-toxin
NKGVCAGD	8	1.3634	Probable Allergen	Non-toxin

### 3.5 Mapping the vaccine construct

**Fig 2:**
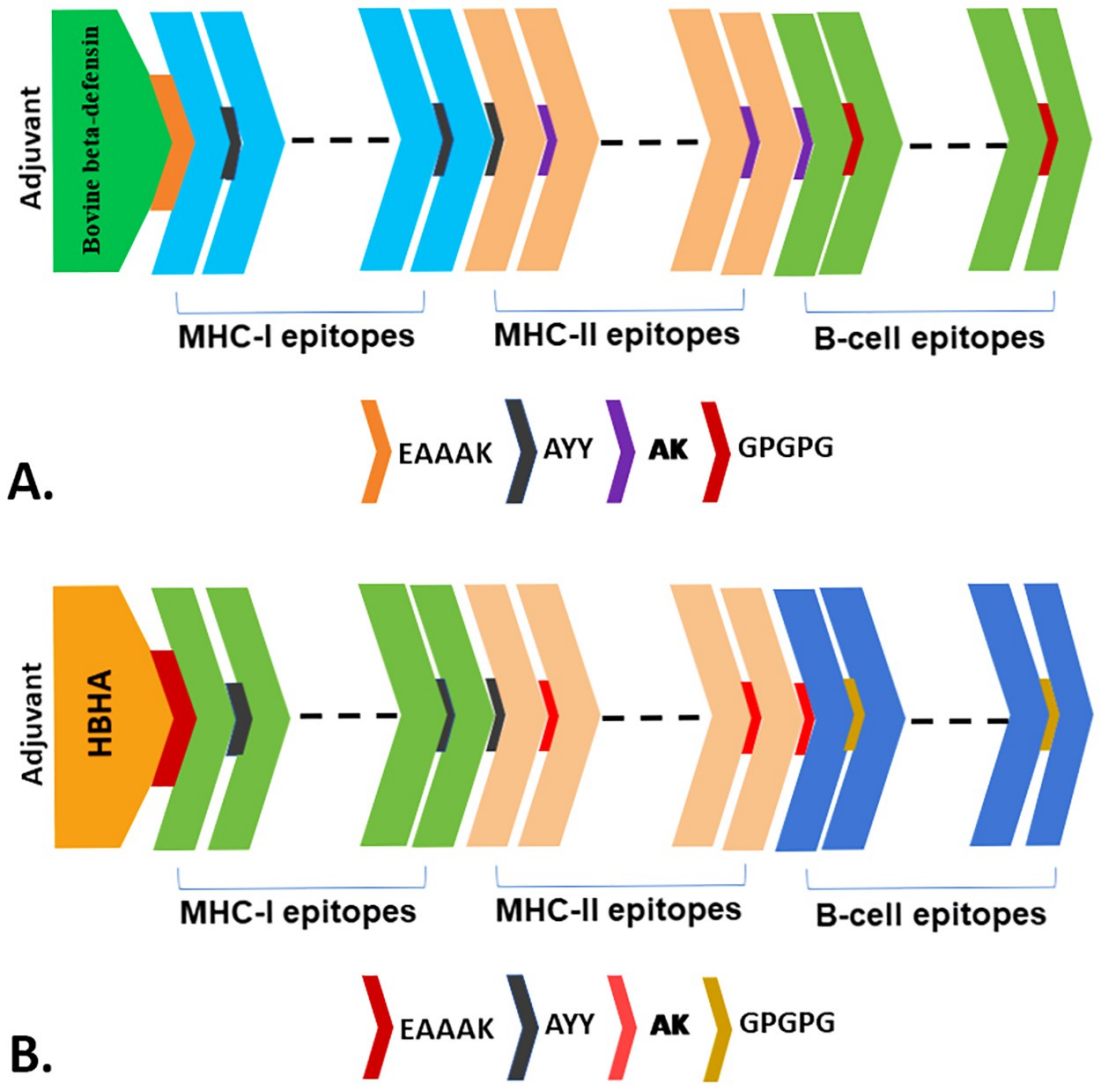
The schematic representation of multiepitope-based vaccine constructs (V1 and V2). The V1 (A) and V2 (B) containing adjuvants, epitopes, and linkers were depicted in different color codes.

Two vaccines, V1 and V2, against *Echinococcus* spp., were developed by selecting the most promising epitopes based on specific criteria such as antigenicity, non-allergenicity, and non-toxicity. The epitopes were conjugated using two adjuvants named bovine beta-defensin and HBHA. Four linkers, EAAAK, AYY, AK, and GPGPG, were used to join the selected epitopes and adjuvants in the final vaccine structure. Adjuvants, a key component in vaccine development, enhance the vaccine’s ability to trigger an immune response and enhance its stability and longevity [93]. The EAAAK linkers connected the adjuvants bovine beta-defensin for V1 and HBHA for V2. After that, the MHC-I epitopes were linked to each other by the AYY linkers. The AAY linker, which conjugates the epitopes that showed high specificity, is frequently used in *in-silico* vaccine design studies [94]. After that, AYY adjoined the MHC-II epitopes by the AK linker and further connected using the GPGPG linkers. The GPGPG linkers may block junctional epitope generation while promoting immune processing and presentation [95]. The GPGPG linkers were used to attach the linear B-cell epitopes to the vaccine construct and link the epitopes together. Finally, the V1 and V2 were built and allowed for further analysis (Fig 2).

### 3.6 Evaluation of physicochemical properties, solubility, allergenicity, and antigenicity of individual vaccine constructs

As physicochemical properties, the ExPASy/ProtPram computed a total number of 324 amino acids in V1 and 390 in V2, while the molecular weights were calculated as 36758.83Da and 42911.49Da, respectively. The server confirmed that the multiepitope vaccines are stable proteins with instability indices of 59.26 and 46.37 for V1 and V2, respectively. The isoelectric point (pI) of the vaccines was calculated as 9.37 (V1) and 6.09 (V2), which inferred that the V1 has basic (pH > 7), while the V2 has acidic (pH < 7) nature. Moreover, the predicted GRAVY scores of the V1 and V2 were -0.507 and -0.433, respectively, indicating that the vaccines are water-soluble (Table 4). On the other hand, both vaccines were effectively soluble upon expression in *E*. *coli*, confirmed by the SOLpro server. Regarding solubility, both vaccines showed solubility with scores of 0.589633 and 0.781243 (threshold value of 0.45) for V1 and V2, respectively. The AllerTOP v. 2.0, the AllergenFP v.1.0, and the AlgPred server predicted no allergenicity in the vaccine; thus, the vaccine is non-allergenic (Table 4). In addition, the Vaxijen and SCRATCH servers revealed that the vaccines acted like probable antigens with a better antigenic score.

**Table 4:** The physicochemical and immunological properties of the vaccine.

Physicochemical properties	V1	V2
Molecular Weight (Da)	36758.83	42911.49
Number of amino acids	324	390
Theoretical isoelectric point (pI)	9.37	6.09
Grand average of hydropathy (GRAVY)	-0.507	-0.433
Aliphatic index	62.62	70.90
Total number of negatively charged residues (Asp+Glu)	28	53
Total number of positively charged residues (Arg+Lys)	41	50
Total Number of atoms	5097	6008
Extinction coefficient (assuming all pairs of Cys residues form cystines)	68800	57425
Extinction coefficient (assuming all Cys residues are reduced)	68300	57300
Instability index	59.26	46.37
Solubility / SOLpro	Solubility upon over-expression (0.589633)	Solubility upon over-expression (0.781243)
Allergenicity/AllerTOP v. 2.0	Probable non-allergen	Probable non-allergen
Allergenicity / AllergenFP v.1.0	Probable non-allergen	Probable non-allergen
Allergenicity/ AlgPred	Non-allergen	Non-allergen
Antigenicity /Vaxijen	Probable antigen	Probable antigen
Antigenicity/ SCRATCH	Probable antigen	Probable antigen

### 3.7 Secondary structure prediction

**Fig 3:**
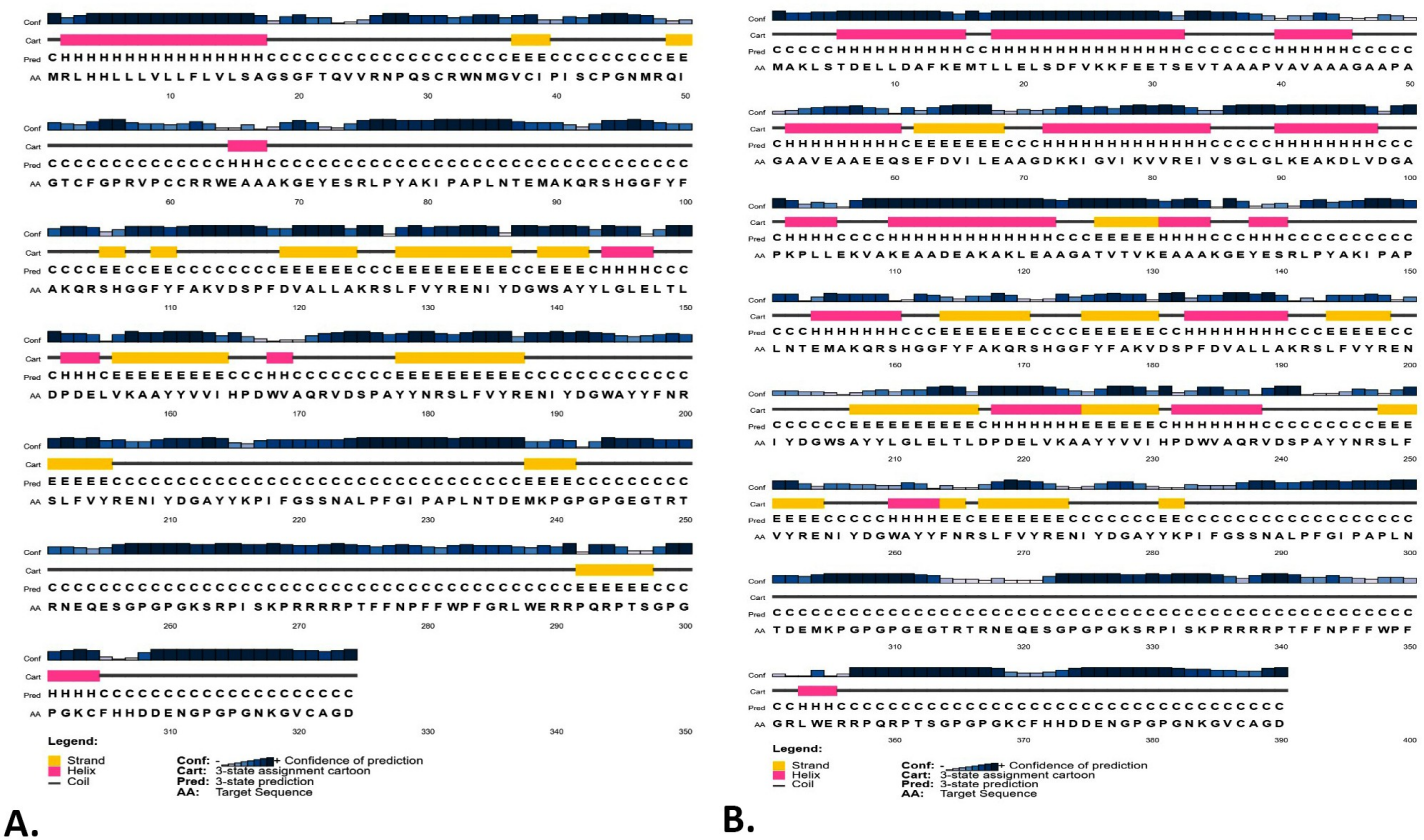
The secondary structures of the V1 (A) and V2 (B) were predicted by the PSIPRED server. The confidence of the prediction is represented by dark blue bars, while the helices, beta-strands, and coils structure are predicted by the pink, yellow, and line bars, respectively.

Based on SOPMA prediction, the secondary structure of the V1 consists of a random coil of 52.78%, alpha helix of 19.44%, and extended strands of 20.68%. The V2 comprises 39.23% random coil, 35.38% alpha helix, and 14.87% extended strands. Similarly, the GOR4 server predicted 64.81% random coil, 14.51% alpha helix, and 20.68% extended strands within the secondary structure of V1. The server predicted 51.28% random coil, 33.85% alpha helix, and 14.87% extended strands within the V2 (S1 Table). The PSIPRED server also offered information on the protein’s secondary structure, predicting three states: coil, helix, and strands (Fig 3, S2 Fig).

### 3.8 Tertiary structure prediction, energy minimization, and structural validation

**Fig 4:**
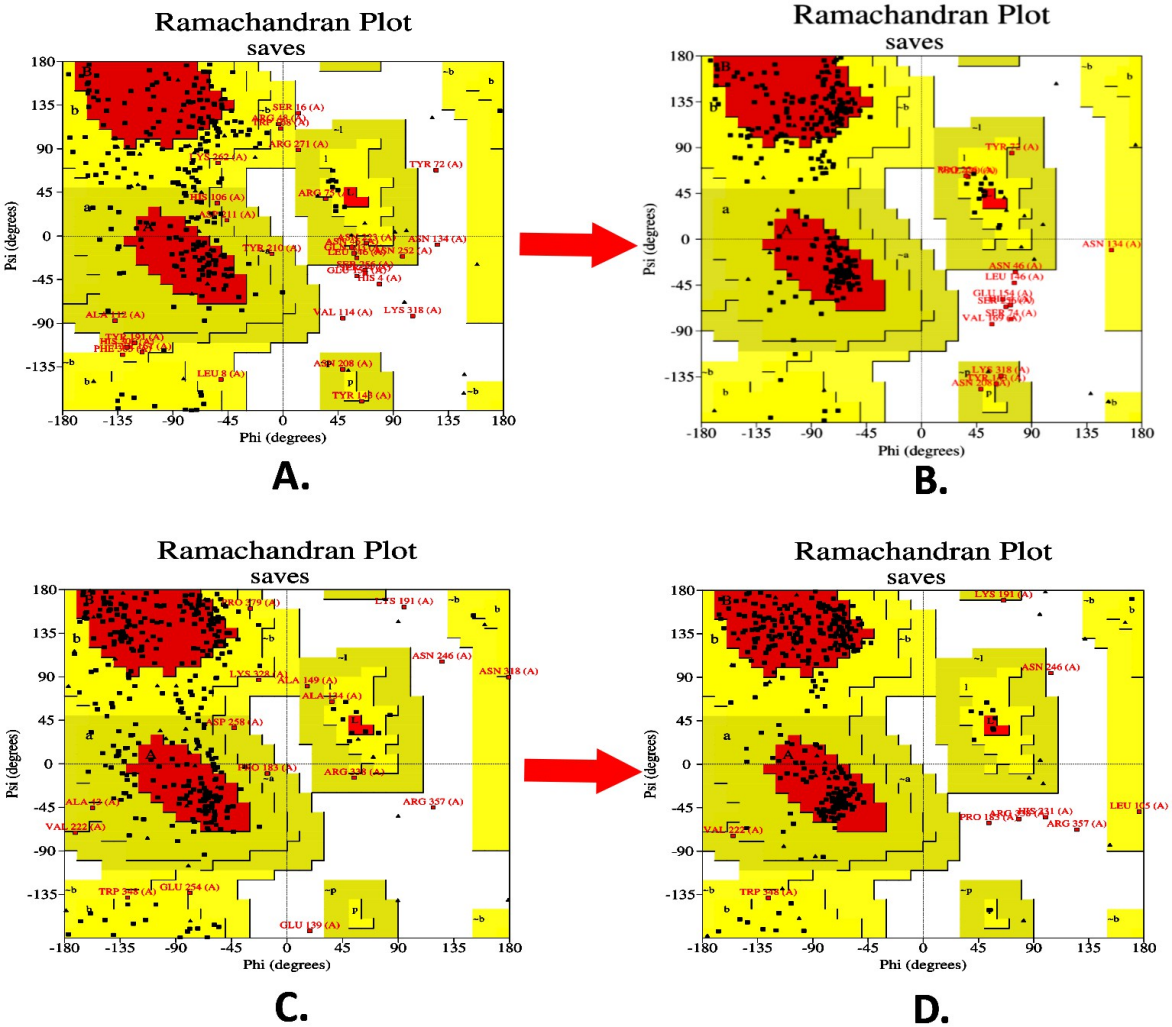
Ramachandran plots of the predicted vaccines: V1 and V2. The plots represent residual quality before (V1: A, V2: C) and after (V1: B, V2:D) the refinement process.

Among all the models predicted by the I-TASSER server, the first model was chosen as it had the highest C-score of -1.02. Additionally, the TM-score was found to be 0.78±0.11, confirming the correct topology of the model. Finally, the RMSD of the predicted model was 8.8±4.4 Å, signifying the structural stability (S3 Fig). Following the prediction of the 3D structure of the V1 and V2, the GALAXYrefine tool of the GALAXYWEB server was employed to refine the structure, improving the accuracy of the vaccines’ 3D models. The Ramachandran plot study revealed that after refinement, the amino acid residues of the V1 were reported in the most favored region, additional allowed regions, generously allowed regions, and disallowed regions with percentages of 69.6%, 25.3%, 2.3%, and 2.7%, respectively. In the case of V2, the percentage of amino acid residues in the most favored region was increased by 82.6%, where, in additional allowed, generously allowed, and disallowed regions, these were reported as 25.3%, 2.3%, and 2.7%, respectively (Table 5; Fig 4). The ERRAT quality factors of V1 and V2 were 39.27 and 58.95, respectively (Table 5).

**Table 5:** The structural validation of the final vaccine construct by SAVES, ProSA, and SWISS-MODEL structure assessment server.

Server	Vaccine	SAVES			ProSA	SWISS-MODEL Structure Assessment	
		PROCHECK (Ramachandran favored region)	ERRAT	VERIFY3D	Z-Score	MolProbity Score	Ramachandran favored region
**Before refinement**	V1	42.2%	65.82	93.83%	-4.71	3.19	49.43
	V2	57.8%	81.87	61.79%	-2.87	2.94	65.69%
**After refinement**	V1	69.6%	39.27	91.05%	-6.59	2.29	86.02%
	V2	82.6%	58.95	57.95%	-5.71	1.90	89.95%

**Fig 5:**
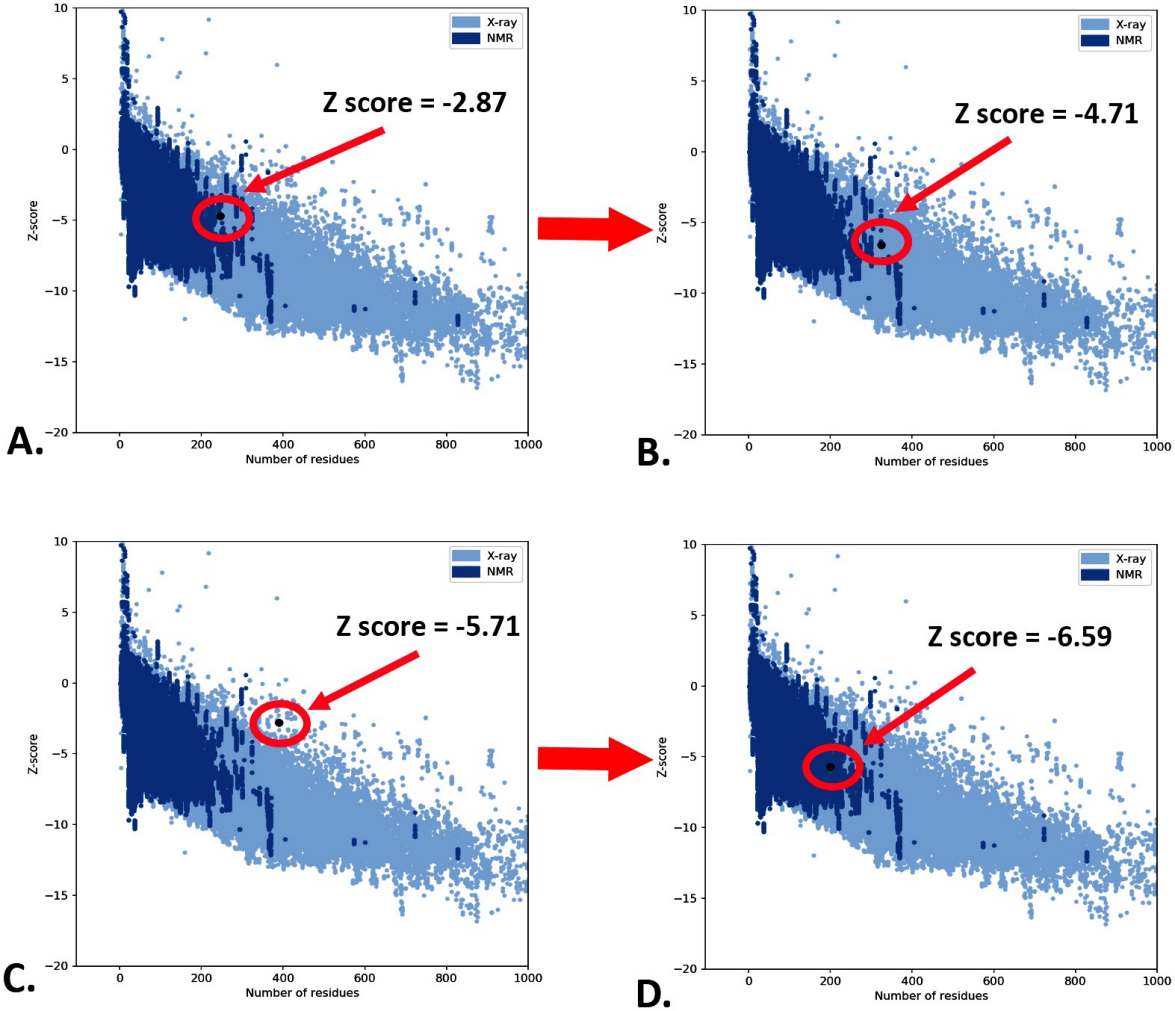
The Z score of the vaccines’ 3D models; before (V1: A, V2: C) and after (V1: B, V2:D) refinement process.

The ProSA server affirmed that the energy-minimized models of the V1 and V2 had the Z-score of -6.59 and -5.71, respectively. The unminimized models of the V1 and V2 had the Z score of -4.71 and -2.87, which indicates the predicted model’s quality accuracy after the refinement process (Table 5; Fig 5). Later, the SWISS-MODEL structure assessment server verified the two refined 3D models of the V1 and V2. Before refining, it was observed that the MolProbity score of V1 and V2 were 3.19 and 2.94, while Ramachandran’s favoured regions were 49.43% and 65.69%, respectively. However, after refinement, the MolProbity score of V1 and V2 were found as 2.29 and 1.90, while Ramachandran’s favored regions were found as 86.02% and 89.95%, respectively (Table 5). However, after analyzing all these structural parameters, we choose V2 for our subsequent analysis. Moreover, the superimposition between the I-TASSER predicted model and GalaxyWEB refined model of the V2 ensured the suitability of the refined model for further proceedings (S4 Fig).

### 3.9 Discontinuous B-cell epitope prediction

**Fig 6:**
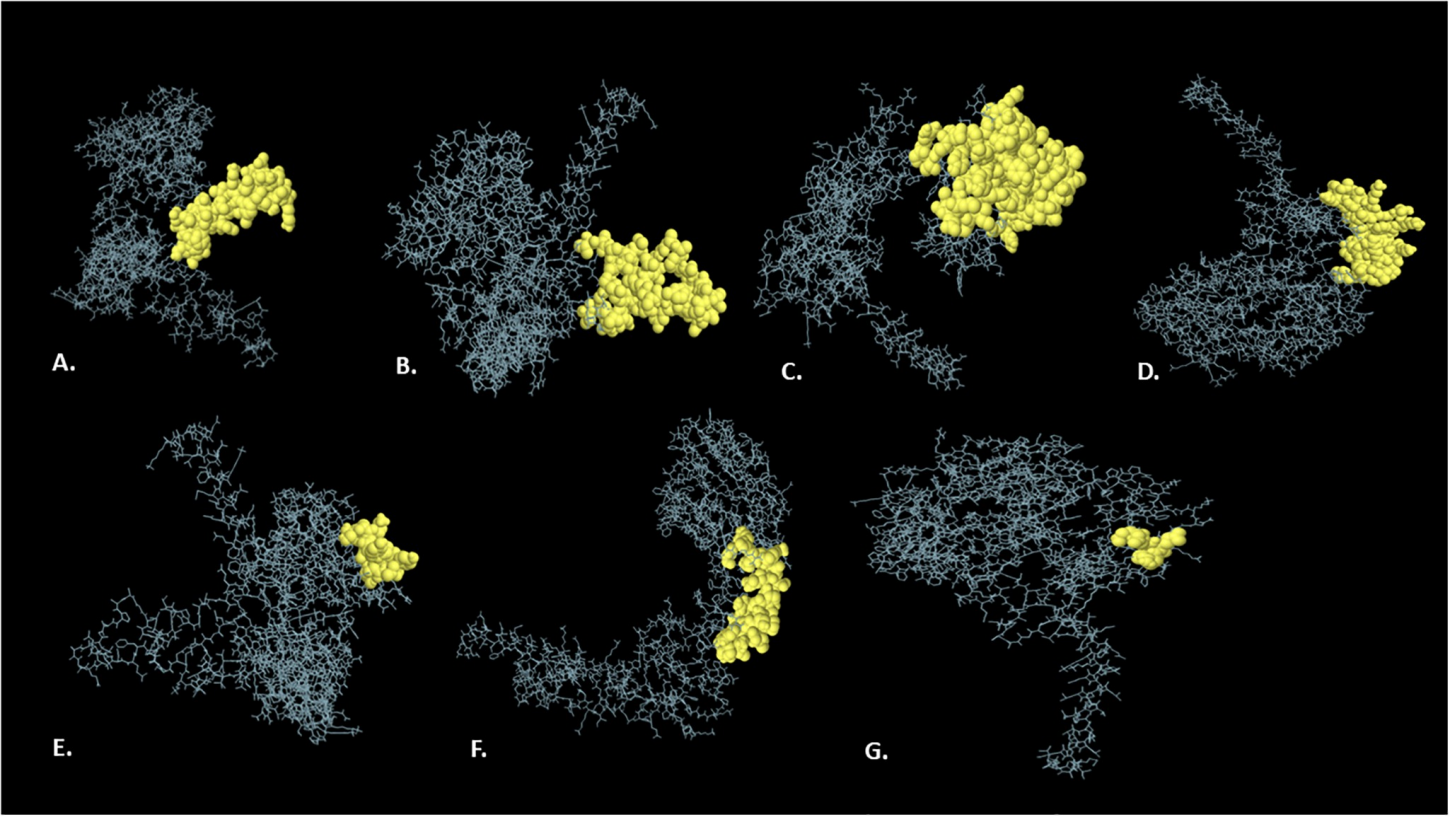
The 3D illustration of predicted discontinuous B-cell epitopes of the V2 (A–G), where the yellow color surfaces indicate the discontinuous B-cell epitopes and the gray sticks display the whole vaccine.

The Ellipro server predicted seven discontinuous B-cell epitopes for the V2, which have 390 amino acid residues in total and scores ranging from 0.512 to 0.873 with individual residues (S2 Table). The higher the score, the more likely the area in the query will serve as a conformational B-cell epitope. Therefore, these regions of the vaccine might act as B-cell epitopes and thus would be effective in generating potential antibodies (Fig 6).

### 3.10 Molecular docking of the vaccine constructs

**Fig 7:**
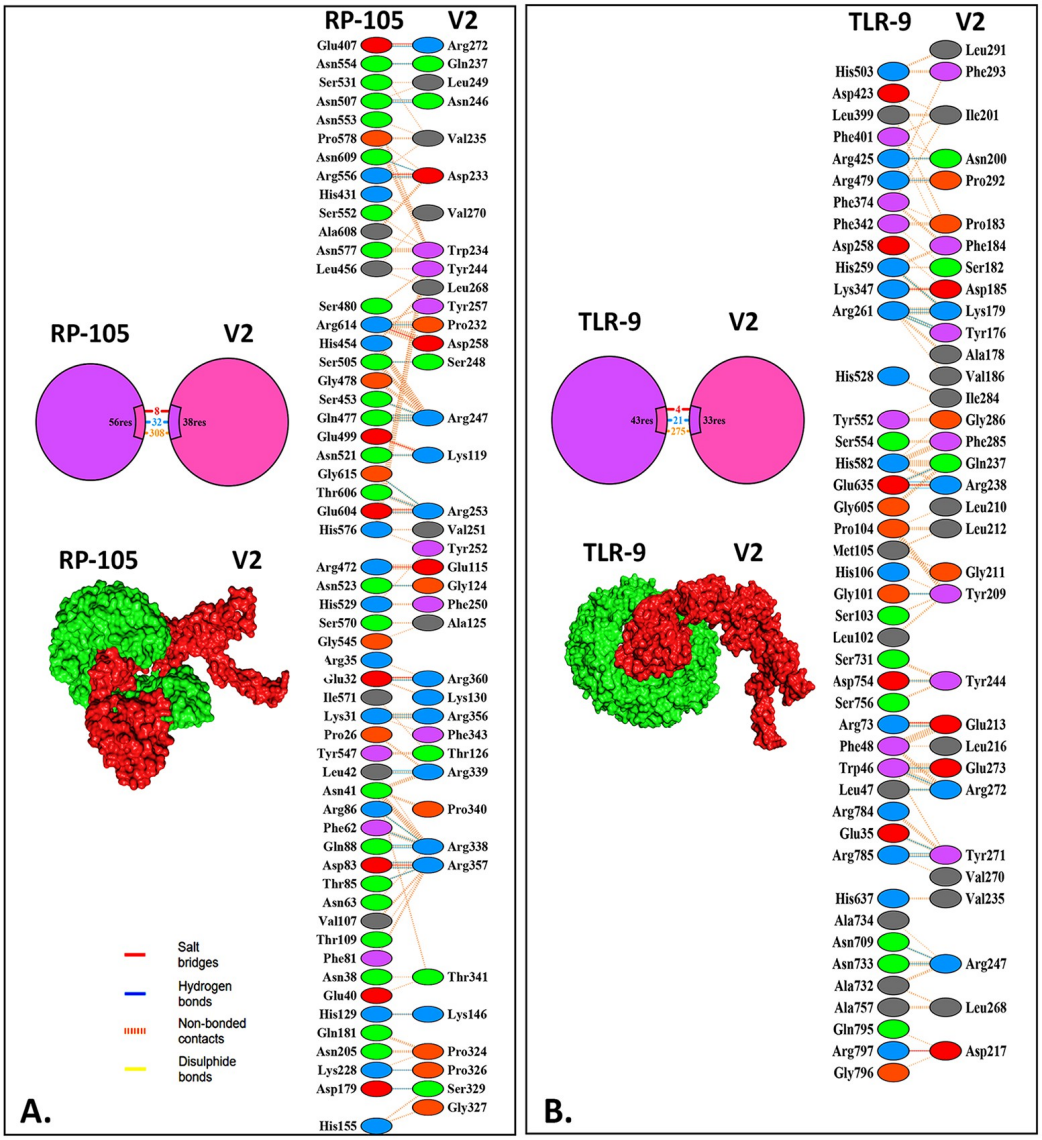
The molecular interactions of the V2-RP-105 and the V2-TLR-9 complexes. The vaccine and receptors are shown in red and green, respectively, with their interactions illustrated using different shades based on bond properties.

Cluspro 2.0 server demonstrated a remarkable affinity of the vaccine towards the immune receptors (RP-105 and TLR-9) during the molecular docking analysis. The best two vaccine-receptor docked complexes were selected based on their global binding energy and electrostatic interaction. However, the V2-RP-105 complex showed a center energy score and lowest energy score of -1252.7 and -1299.3, respectively. Besides, the V2-TLR-9 complex showed a center energy score and lowest energy score of -1266.8 and -1290.2, respectively (Table 6; Fig 7). Further, PyMOL and PDBsum visualized and analyzed these docked complexes. According to the PDBsum, the V2-RP-105 complex has 32 hydrogen bonds, 8 salt bridges, and 308 non-bond interactions (Table 6; Fig 7). Meanwhile, the V2-TLR-9 complex has 21 hydrogen bonds, 4 salt bridges, and 275 non-bond interactions.

**Table 6:** The molecular docking score of the V2-RP-105 and the V2-TLR-9 with their molecular interactions.

Complex	Weighted Score		Interactions between the vaccine and TLRs			
	Center	Lowest energy	Salt bridges	Disulphide bonds	Hydrogen bonds	Non-bonded contacts
V2-RP-105	-1252.7	-1299.3	8	-	32	308
V2-TLR-9	-970.9	-1038.5	4	-	21	275

### 3.11 Molecular dynamic simulation

**Fig 8:**
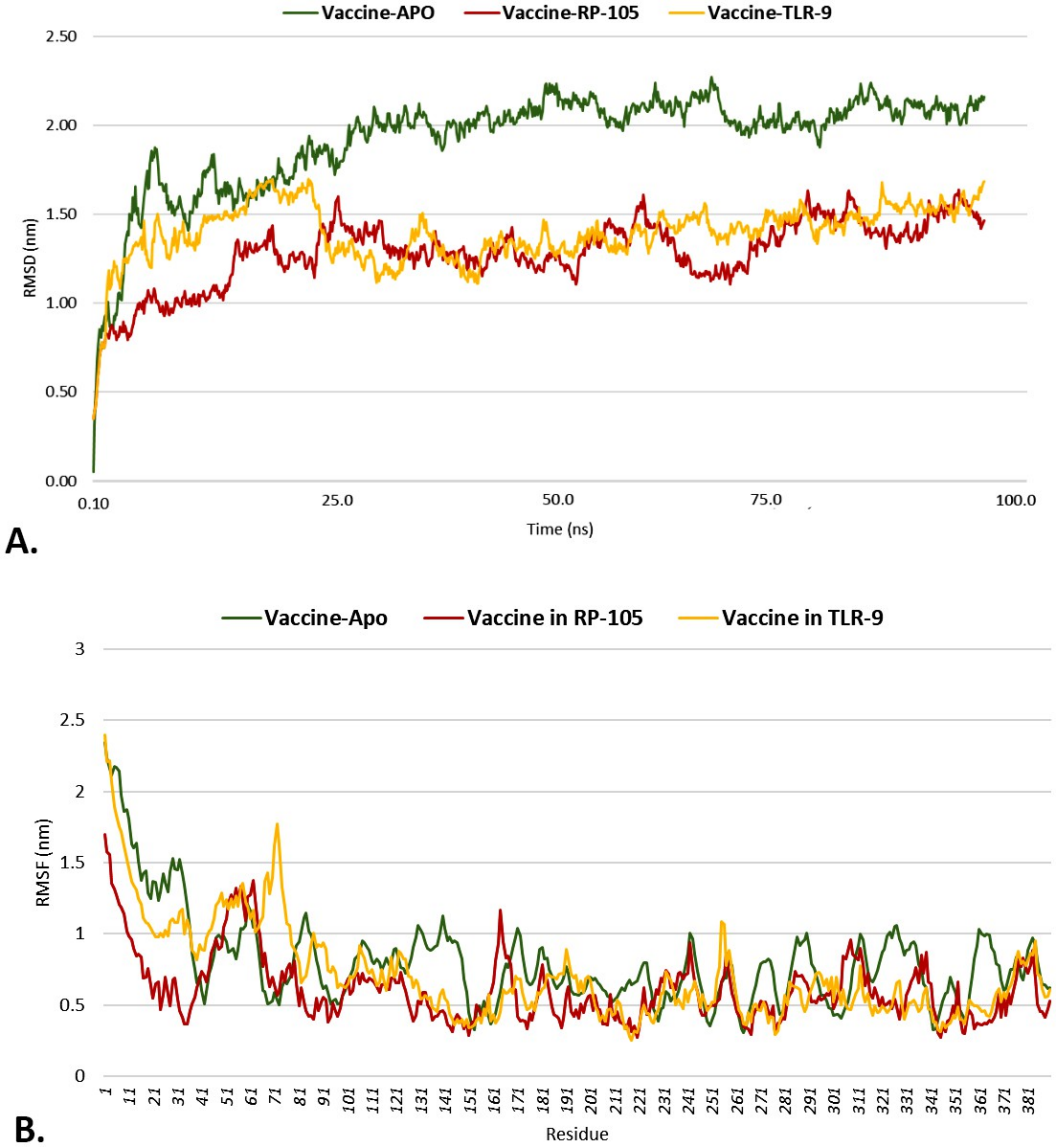
Graphical representation of the molecular dynamic simulation study. The RMSD (A) and RMSF (B) of the V2-apo, V2-RP-105, and V2-TLR-9 were depicted in green, maroon, and yellow colors.

**Fig 9:**
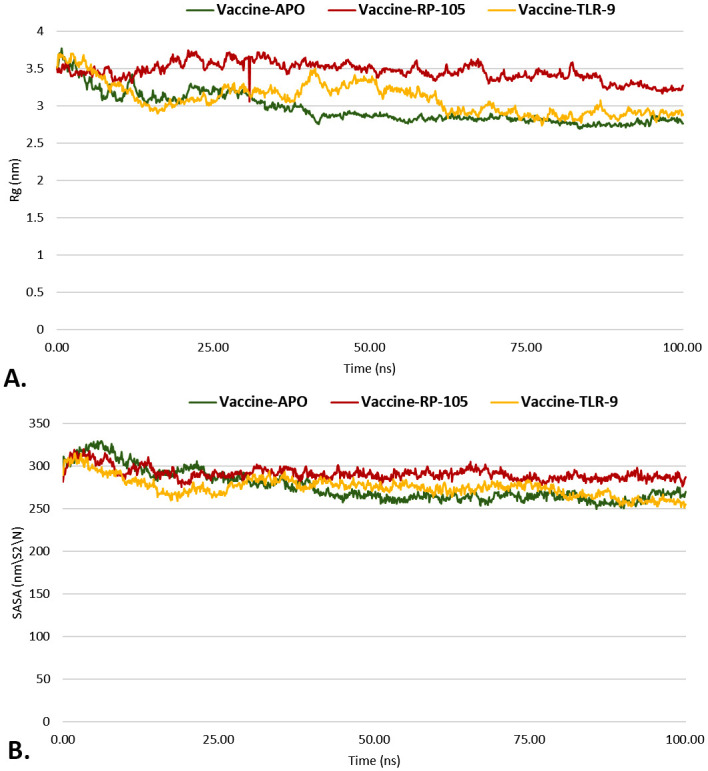
The line plot represents the molecular dynamic simulation study. The Rg (A) and SASA (B) of the V2-apo, V2-RP-105, and V2-TLR-9 were depicted in green, maroon, and yellow colors.

The V2-apo, V2-RP-105, and V2-TLR-9 complexes were dynamically simulated for 100 ns to establish the complex’s stability in dynamic mode. Several assessments, including RMSD, RMSF, Rg, and SASA, were performed for MD simulation. RMSD is frequently applied when analyzing the dynamics and structures of macromolecules. The pattern of the RMSD plot provides insight into two fundamental parameters: (a) whether the system has achieved equilibrium and (b) whether the simulation duration was enough. In these circumstances, the simulation time is adequate for the V2-apo, V2-RP-105 and V2-TLR-9 complexes, as shown by the simulated complexes’ RMSD diagram reaching a plateau condition that validates the system’s equilibration. After 100ns simulation time, V2-apo showed relatively higher RMSD than the V2-RP-105, and V2-TLR-9 complexes (Fig 8A). Also, the ligand-receptor combination is stable in its dynamic state if the RMSD plot shows no dramatic fluctuations. By using RMSF, the variation of V2-apo, V2-RP-105, and V2-TLR-9 complexes were examined. After 100ns simulation time, the RMSF of the V2-apo, V2-RP-105, and V2-TLR-9 complexes were calculated to be an RMSF below 3Å (Fig 8B). The Rg is a metric used to assess the density of a protein. A consistent Rg number indicates a protein’s stable folding, whereas a greater Rg profile signifies less stiffness in the biological system. The Rg values of the V2-apo, V2-RP-105, and V2-TLR-9 complexes fluctuated steadily over time, as seen in, but the values declined after 100ns simulation time (Fig 9A). SASA is employed in molecular dynamic simulations to forecast how much a protein’s hydrophobic core is exposed to solvents. Higher SASA values indicate that a large portion of the protein is in contact with water. In contrast, lower values suggest that most of the protein is inside the hydrophobic core. After 100ns, the SASA values of the V2-apo, V2-RP-105, and V2-TLR-9 complexes were consistent and did not exhibit any significant variations throughout the simulation, which led to a further decrease in interest (Fig 9B).

### 3.12 Free energy calculation by MM-PBSA and MM-GBSA approaches

In the context of the V2-RP-105 and V2-TLR-9 complexes, the ΔTOTAL values, representing the total binding free energy, are important. For the V2-RP-105 complex, the ΔTOTAL values were calculated to be -49.22 kcal/mol in MM-PBSA and -117.38 kcal/mol in MM-GBSA analysis. Similarly, for the V2-TLR-9 complex, the ΔTOTAL values were calculated to be -0.17 kcal/mol and -169.86 kcal/mol in MM-PBSA and MM-GBSA, respectively (Table 7). These negative ΔTOTAL values indicated that the binding processes are spontaneous and the vaccine could favorably bind to the target protein receptors. According to MM-PBSA, the ΔEEL value of the V2-RP-105 was calculated to be -504.90 kcal/mol, but in MM-GBSA, it was estimated to be -984.03 kcal/mol. Based on the MM-PBSA, the ΔVDWAALS, ΔEGB and ΔESURF of the V2-RP-105 were calculated to be -153.47 kcal/mol, 629.01 kcal/mol and -19.86 kcal/mol, respectively. At the same time, the MM-GBSA assay calculated the ΔVDWAALS, ΔEGB, and ΔESURF as -244.0 kcal/mol, 1142.64 kcal/mol, and -31.95 kcal/mol, respectively (Table 7). With the MM-PBSA, the ΔEEL value of the V2-TLR-9 was estimated to be -356.94 kcal/mol, while the MM-GBSA estimated a ΔEEL value of -985.1 kcal/mol. Following the MM-PBSA analysis, the ΔVDWAALS, ΔEGB, and ΔESURF values of the V2-TLR-9 were determined to be -0.28 kcal/mol, 357.06 kcal/mol, and 0.00 kcal/mol, respectively. In comparison, the MM-GBSA analysis provided the ΔVDWAALS, ΔEGB, and ΔESURF values of -228.68 kcal/mol, 1074.7 kcal/mol, and -30.79 kcal/mol, respectively (Table 7).

**Table 7:** The free binding energy calculations of the V2-RP-105 and V2-TLR-9 complexes.

Complex	MM-PBSA					MMGBSA				
	ΔTOTAL (kcal/mol)	ΔEEL	ΔVDWAALS	ΔEGB	ΔESURF	ΔTOTAL (kcal/mol)	ΔEEL	ΔVDWAALS	ΔEGB	ΔESURF
**V2-RP-105**	-49.22	-504.90	-153.47	629.01	-19.86	-117.38	-984.03	-244.0	1142.64	-31.95
**V2-TLR-9**	-0.17	-356.94	-0.28	357.06	0.00	-169.86	-985.1	-228.68	1074.7	-30.79

### 3.13 Normal mode analysis (NMA) of the vaccine-docked complex

**Fig 10:**
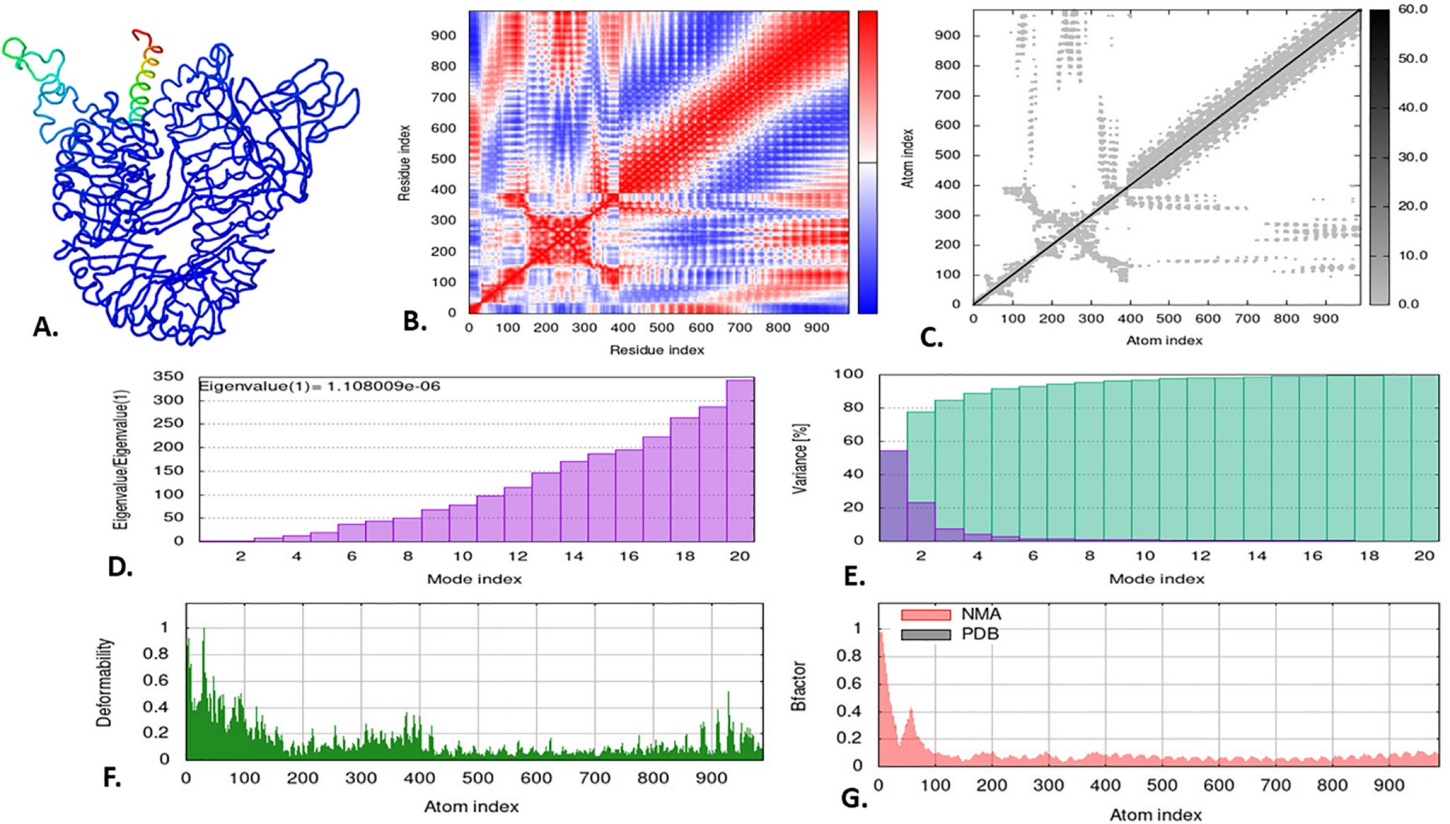
The normal mode analysis of the V2-RP-105 docked complex. The illustration depicted (A) the domain motion of the docked 3D structure, (B) the co-variance map, (C) the elastic network, (D) the eigenvalue, and (E) the variance.

**Fig 11:**
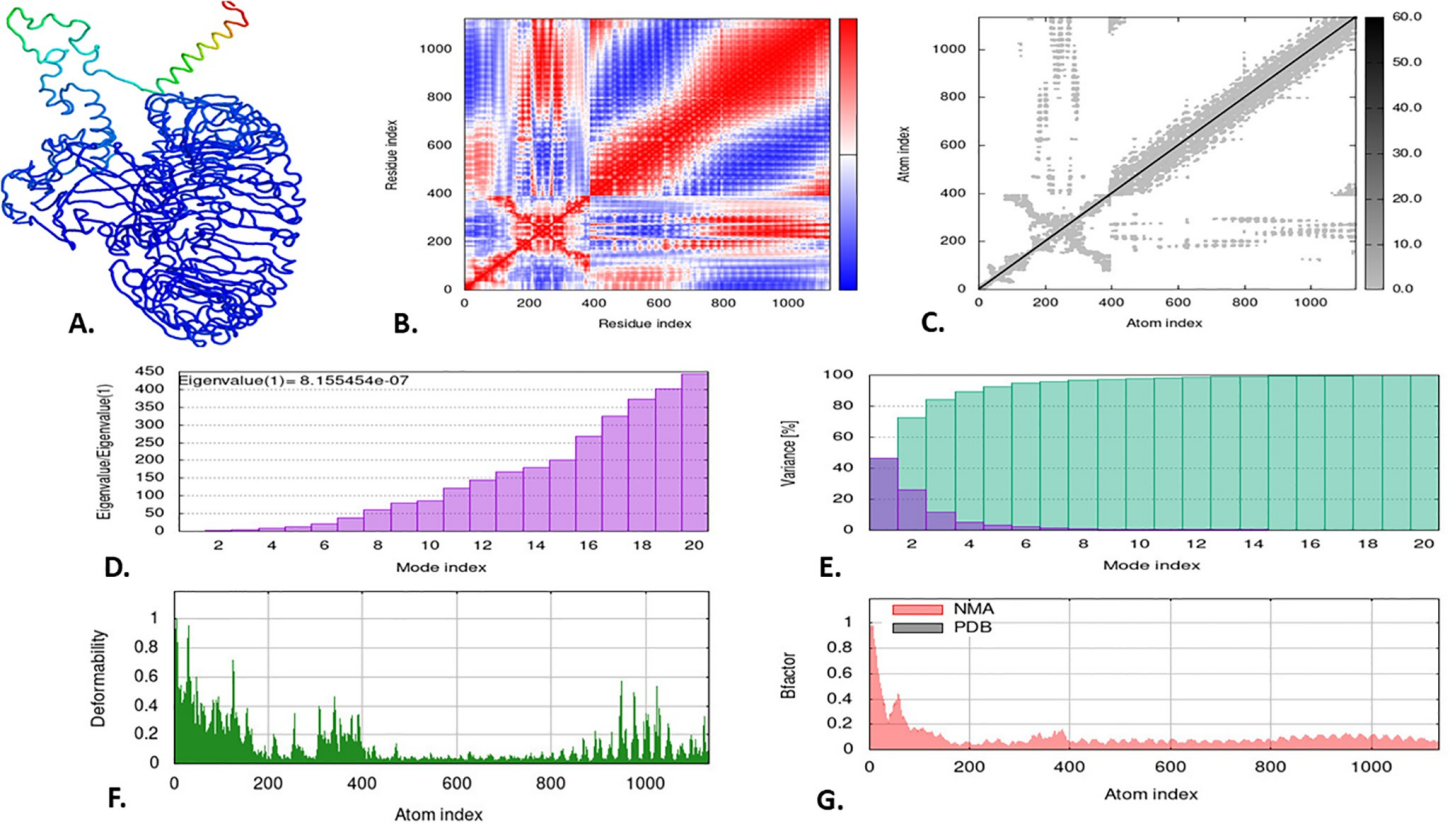
The normal mode analysis of the V2-TLR-9 docked complex. The illustration depicted (A) the domain motion of the docked 3D structure, (B) the co-variance map, (C) the elastic network, (D) the eigenvalue, and (E) the variance.

The iMODS server, renowned for its precision, was utilized to conduct NMA analyses of the V2-RP-105 and V2-TLR-9 docked complexes, providing a comprehensive assessment of their structural integrity and alterations. The movement of these complexes within their domain is vividly depicted as ribbon 3D structures (Figs 10B & 11B). The covariance map in NMA analysis, represented by red, white, and blue colors, illustrates the correlated, uncorrelated, and anti-correlated shifts between residue pairs of the V2-RP-105 and V2-TLR-9 docked complexes (Figs 10B & 11B). The elastic map of the docked complexes, with its darker grey patches, indicates the interaction between the atoms, suggesting the presence of more rigid places (Figs 10C & 11C). An eigenvalue quantifies the influence of individual deformation movements on the overall motion of the protein. A higher eigenvalue indicates substantial displacement, while a lower eigenvalue correlates to collective conformational changes in the protein complex [32, 96]. Furthermore, the eigenvalue of the complexes indicated the structural stability of the V2-RP-105 and V2-TLR-9 docked complexes with the lowest eigenvalue of 1.108009e-06 and 8.155454e-07, respectively (Figs 10D & 11D). When determining molecular flexibility using NMA, the amplitude of each mode’s fluctuation is inversely proportional to the eigenvalue. This relationship is quantitatively expressed as variance [97]. The variance graph depicted the cumulative variance in cyan and the individual variance in purple. Within both docked complexes, the first 3 modes, out of 20 modes, contribute to 80% of the attained variance (Figs 10E & 11E). The deformability graph illustrates the flexible areas of the docked complexes using graphical peaks, where the V2-TLR-9 complex showed higher deformability than the V2-RP-105 complex (Figs 10F & 11F). Finally, the B-factor graph displayed the simulation of the docked complexes involving NMA and the PDB sector. The B-factor values indicate the amplitude of atomic displacements, revealing higher deformability in the V2-RP-105 and V2-TLR-9 docked complexes, suggesting greater flexibility (Figs 10G & 11G).

### 3.14 Codon optimization and *in-silico* cloning

**Fig 12:**
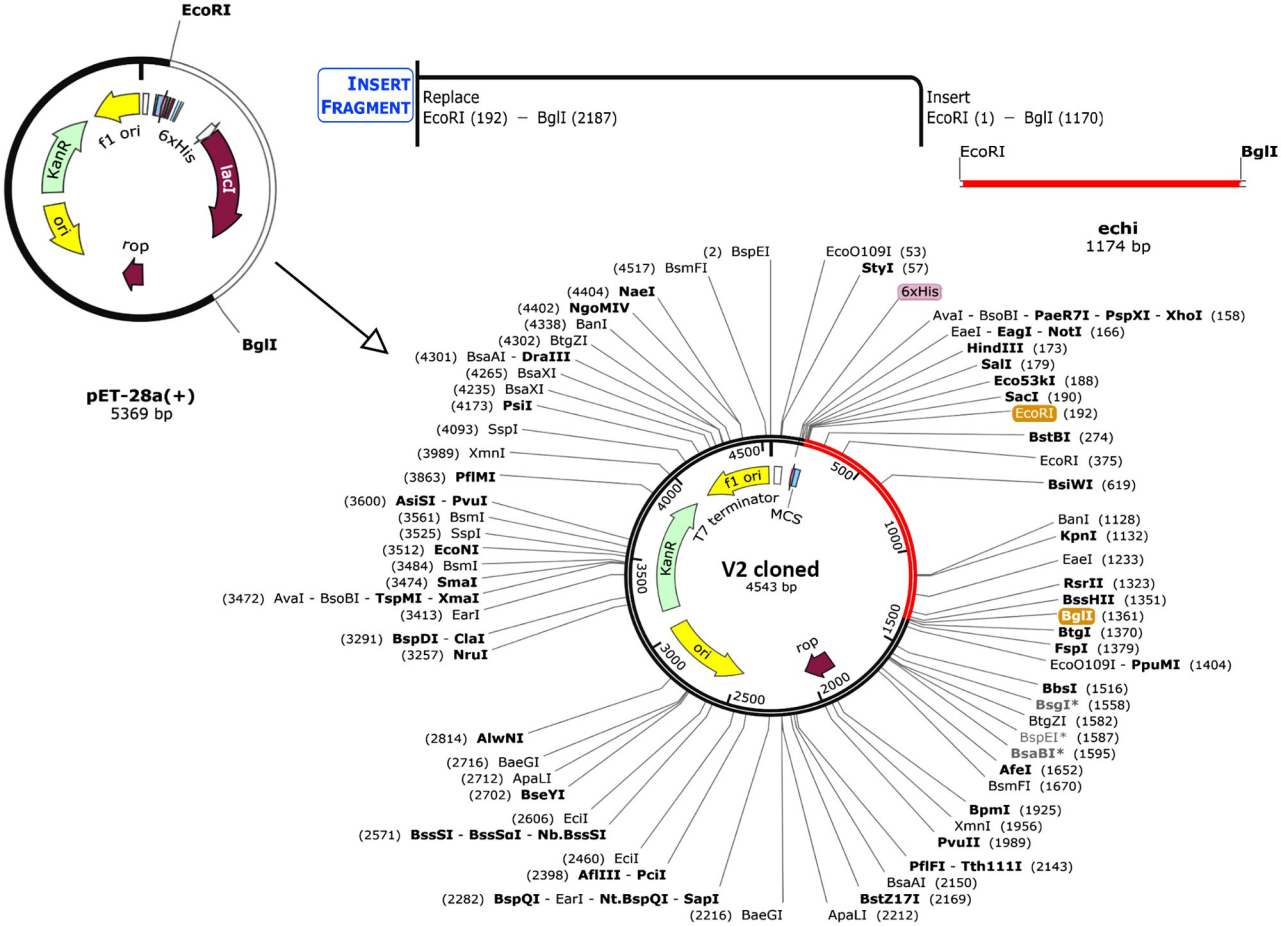
*In-silico* cloning vector cloning of the V2. The pET-28a (+) plasmid vector was modified with the V2 sequence using the SnapGene software free-trial. The vaccine’s gene coding is represented by the red part, while the vector backbone is represented by the black circle.

The JCat server generated an optimal codon sequence that is 2650 nucleotides long. In addition, the codon optimization index (CAI) value and the average GC content of the adapted sequence were determined to be 1.0 and 53.36%, respectively. These findings indicated that the *E*. *coli* host could achieve optimal protein production. Using SnapGene software, the modified codon sequences were inserted into the plasmid vector pET-28a (+) to create a recombinant plasmid sequence (Fig 12).

### 3.15 Immune simulation

**Fig 13:**
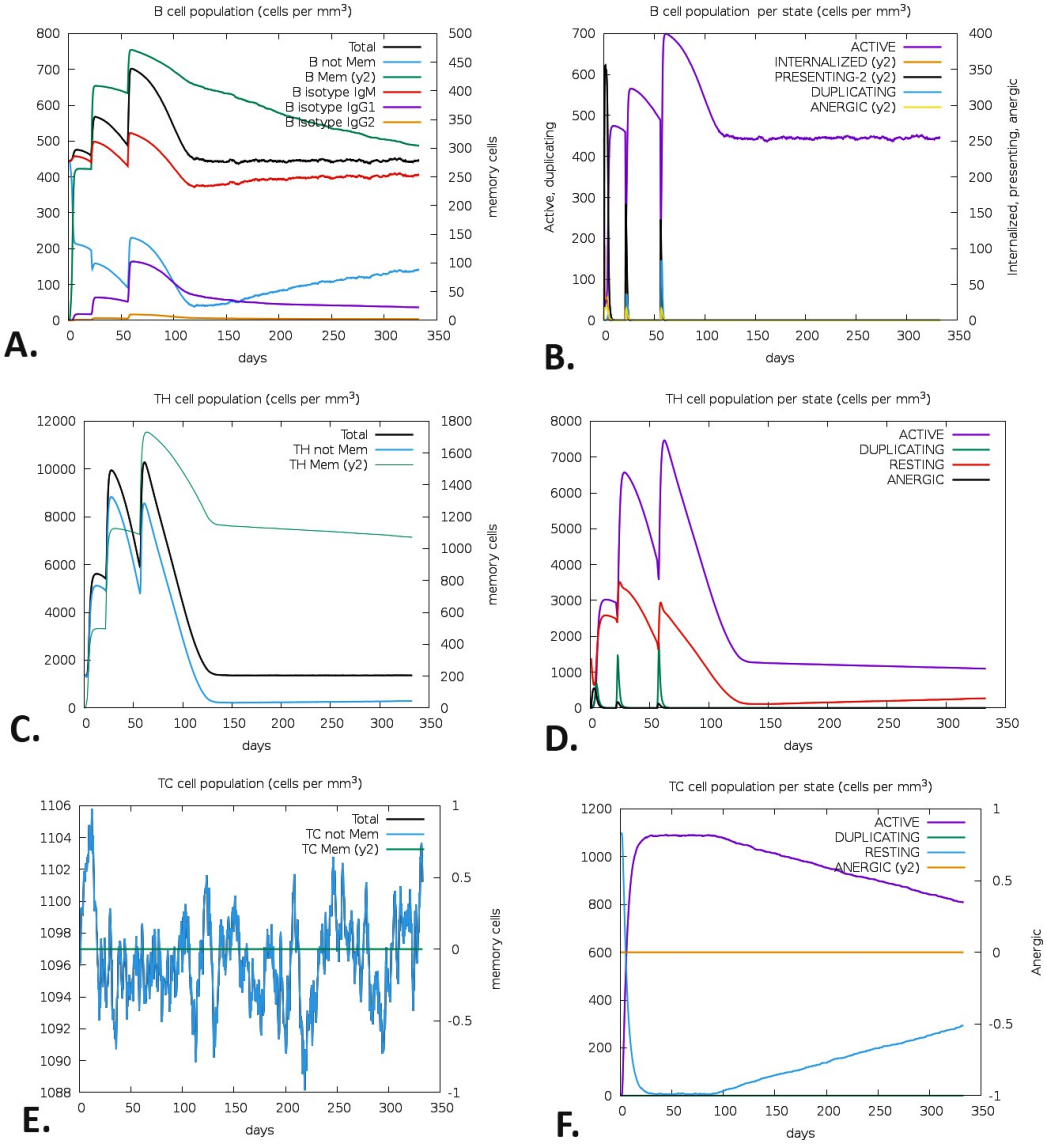
C-ImmSim was used to simulate the V2’s immunological response. The development of B-cell populations (A, B), TH-cell populations (C, D), and TC-cell populations (E, F) after three consecutive injections.

Following immunization on the 1st, 28th, and 56th days, B-cells were significantly increased, particularly memory B-cells (Fig 13A). The immunity produced by each B-cell proved to be long-lasting and lasted for almost a year (Fig 13B). A high level of expression suggested the early immunological responses. Furthermore, the vaccination also activated T cells, namely T helper cells (TH cells) and cytotoxic T cells (TC cells). The study also identified a significant increase in the expression of memory TH and active TH cells at day 60 of post-vaccination, which subsequently decreased over time (Fig 13C & 13D).

**Fig 14:**
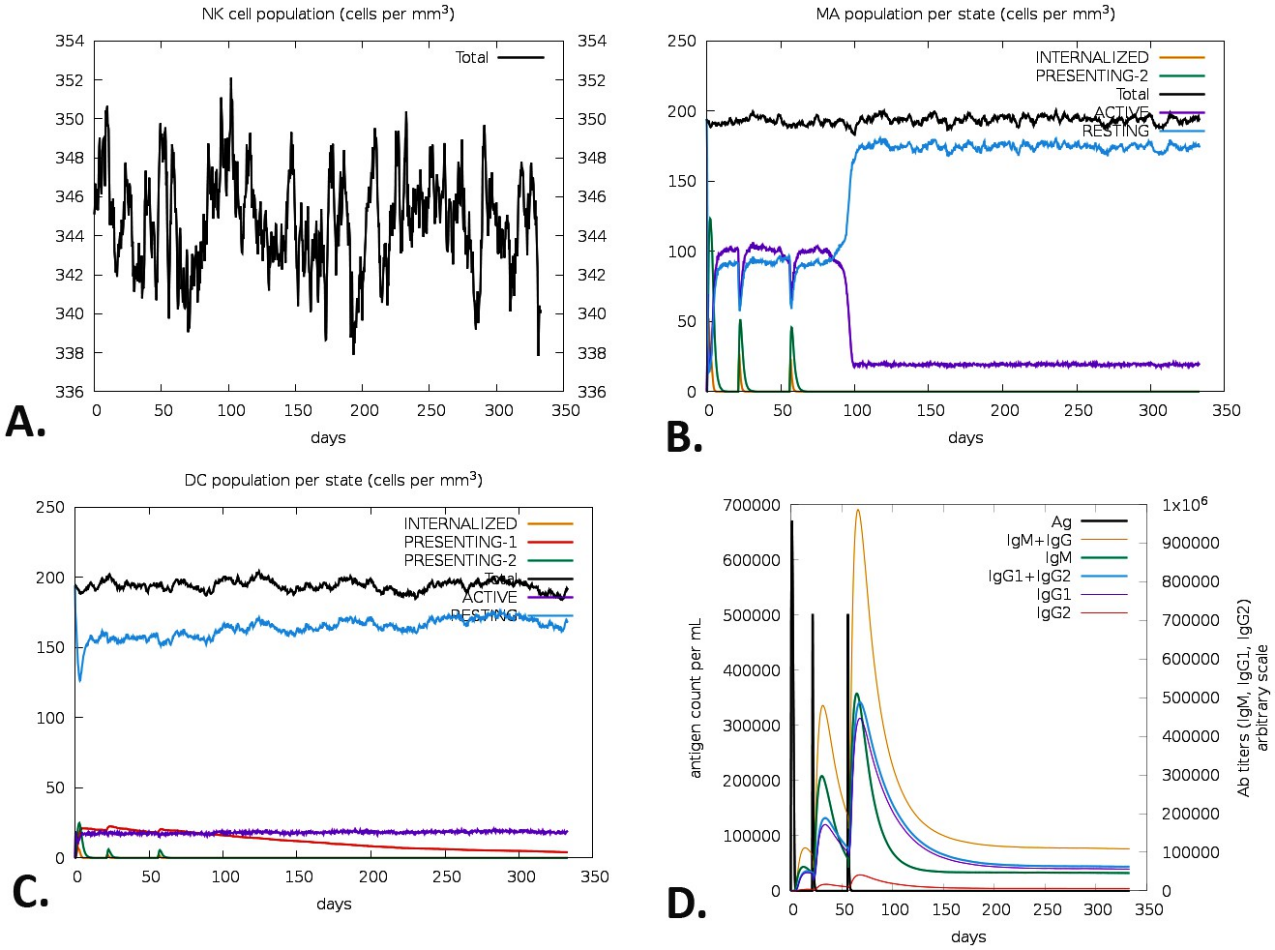
C-ImmSim predicted innate immune responses by the V2. The evolution of (A) NK cells, (B) MA cells, (C) DC-cells, and (D) humoral and cytokine responses by the V2.

Moreover, the expression of memory cytotoxic T cells (TC) and active TC cells was at its highest level and persisted for a very extended duration (Fig 13E & 13F). The innate immune responses were assessed after the post-vaccination regimen, explicitly focusing on the levels of natural killer (NK) cells (Fig 14A), macrophage (MA) (Fig 14B), and dendritic cells (DC) (Fig 14C). Following a peak at day 60 of post-vaccination, the expression of IgM+IgG steadily decreased, indicating the strength and durability of the initial immune response. Following vaccination, the immune response remained effective for two months. The trials demonstrated significant production of IFN-γ (Fig 14D).

## 4. Discussion

Echinococcosis has been reported in both humans and livestock, and even in wildlife [32], and it poses a global public health threat [32, 96]. The true pathogen, especially *Echinococcus* spp., has extensive genetic diversity, with distinct strains exhibiting variations in pathology and drug responses [11]. Preventing the disease requires the development of specific and compelling therapeutics for community protection, particularly in developing countries, while the vaccine is still the first priority [15]. "Systems vaccinology" and "immunoinformatics" are two efficient and precise mathematical and computational techniques that can reduce the time and expenses of vaccine development [97].

Immunoinformatics provides a revolutionary technology for developing vaccines with several benefits. To begin with, it uses machine learning algorithms to swiftly sort through massive amounts of structural, genomic, and proteomic data to find possible vaccine candidates [26]. Immunoinformatics allows for the prediction of immunogenic epitopes and antigenic targets, which improves the process of choosing vaccine components with improved safety and efficacy. This precision-driven approach minimizes the need for laborious and costly empirical testing, optimizing resources and expediting vaccine development timelines [26, 98]. Additionally, immunoinformatics allows for the development of customized vaccines to target certain pathogen strains or host populations, increasing their efficacy and versatility in different circumstances. Furthermore, this approach fosters innovation by facilitating the exploration of novel vaccine modalities, such as nucleic acid and viral vector vaccine [98, 99]. However, several issues must be overcome when translating immunoinformatics into manufacturing vaccines. Immunoinformatics depends on extensive data sets, yet the accuracy of predictions for vaccine targets may be affected by data quality and quantity variations. In addition, predicted targets may not consistently generate the intended immune responses or may incite unfavorable reactions [98, 99]. The presence of a wide range of genetic variations in the host population, together with the continuous development of pathogens, adds complexity to the circumstances and may gradually reduce the effectiveness of vaccines. Regulatory approval, production scalability, and cost-effectiveness pose additional challenges, requiring extensive resources and expertise [98, 99]. Addressing these challenges demands interdisciplinary collaboration, technological advancements, and sustained research efforts to realize the promise of immunoinformatics in vaccine development. However, several multiepitope vaccines have been designed against *Echinococcus* spp. using different potential antigens including Eg95, EgA31, EgG1Y162, EgTeg, EgFABP1, EmEMY162, EmLAP, EmGLUT1 and Em-TSP3 [15, 97].

In various host species and countries, the Eg95 hydatid vaccine has demonstrated remarkable efficacy and reliance in eliciting specific immune responses against echinococcosis since 1996 [100, 101]. According to a recent study, the Eg95 protein-based vaccine showed potent and significant antigenicity and was commercially available for use in sheep [102, 103]. This vaccine also demonstrated significant efficacy and reliability in experiments conducted in China, Romania, Argentina, New Zealand, Australia, China, Chile, and Iran [104]. According to another study, the Eg95 vaccine against cattle’s echinococcosis had a protection level of 89 to 99% [101], while the yearly booster dose provided five years of protective immunity [100]. Utilizing immunoinformatics, Nourmohammadi et al., (2020) and Zhao et al., (2019) designed two multiepitope vaccines for the CE targeting the antigenic protein EgA31and EgG1Y162 of *E*. *granulosus*. These vaccines showed promising outcomes despite the lack of pre-clinical and clinical trials [32, 97]. Yu et al., (2021) designed another multiepitope vaccine for the CE using the EgTeg and EgFABP1 proteins of *E*. *granulosus*. However, the vaccine was tested in both *in vivo* and *in vitro* experiments, and it showed potent antigenicity and immunogenicity [16]. Using the potent antigenic protein Em-EMY162 and Em-TSP3 of *E*. *multilocularis*, Li et al. (2022) developed a multiepitope vaccine for the AE, which was claimed to be an effective prophylactic or therapeutic agent against echinococcosis infection [31]. Moreover, Zhou et al., (2023) [30] designed another multiepitope vaccine using the EMY162, LAP, and GLUT1 proteins of *E*. *multilocularis*. The vaccine showed remarkable immunogenicity and antigenicity in mice model [30]. However, these vaccines failed to concurrently target the CE and AE or the two most common species of *Echinococcus*, *E*. *granulosus* and *E*. *multilocularis*. In this study, we designed a multiepitope vaccine against the CE and AE utilizing the Ag5 protein of both *E*. *granulosus* and *E*. *multilocularis* for the first time. Ag5 is an essential antigen that exhibits strong antigenicity in both the definitive and intermediate host, including humans. The glycosaminoglycan-binding motif of the protein might help conserve itself in the host tissue surrounding the parasite [27, 105]. Additionally, MHC-I molecules serve as immunological stimulants for the immune system and have the potential to elicit CD8+ T cell responses against intracellular pathogens. On the other hand, MHC-II molecules are crucial in presenting immunogenic processed peptides to the T cell receptor (TCR) located on CD4+ T cells [89, 106, 107]. The selected MHC-I and MHC-II epitopes of the Ag5 were reported as solid binders and exhibited a notable level of antigenicity without evidence of allergenicity and toxicity. The predicted B-cell epitopes were also highly antigenic, with no evidence of allergenicity. In addition, B-cell epitopes are widely recognized as a crucial component in vaccine construction since they significantly contribute to antigen-antibody interactions [89]. After that, we used four linkers and two adjuvants to construct the vaccines. The developed epitope vaccines also had high antigenicity with no evidence of allergenicity. The designed V1 and V2 were estimated to have a molecular weight (MW) of 36758.83Da and 42911.49Da, respectively, and were found to be highly soluble after being expressed. The solubility of a recombinant protein is determined in over-expressed *E*. *coli* and is critical in the *in-silico* vaccine construct [108]. The vaccines’ theoretical isoelectric point, instability index, and aliphatic index indicated that the vaccines possess hydrophobic properties, aligning with reported aliphatic side chains. When developing an effective vaccine, it is crucial to understand the way a protein folds into its secondary and tertiary structures [55, 57, 64]. The refining process of the V1 and V2 increased the stability of the tertiary structures. These data indicated that the overall quality of the V2 model may be considered satisfactory. Therefore, we chose the V2 for the subsequent analysis. The docking study utilized RP-105 and TLR-9 to assess the possible binding association between the V2 and the immune receptors. The docking analysis confirmed that the V2 had a significant affinity towards the RP-105 and TLR-9. The stability and dynamic performances of the V2-apo, V2-RP-105, and V2-TLR-9 complexes were evaluated by a 100ns simulation run. The simulation time was deemed sufficient for V2-apo, V2-RP-105, and V2-TLR-9 complexes, evidenced by RMSD reaching a plateau, indicating equilibration. However, V2-apo exhibits higher RMSD in post-100ns simulation run than other complexes, suggesting the dynamic stability of the V2-RP-105 and V2-TLR-9. The RMSF analysis shows all complexes maintain RMSF below 1 nm in post-100ns, indicating minimal residue fluctuations. Rg values fluctuated initially but declined in the post-100ns, suggesting stabilized folding. Consistent SASA values in the post-100ns imply sustained hydrophobic core shielding. Overall, these metrics confirmed the stability and equilibration of the complexes within the simulation timeframe.

The obtained negative ΔTOTAL values from both MM-PBSA and MM-GBSA analyses indicated that the binding process between the vaccine and receptors is thermodynamically favorable and spontaneous. The ΔTOTAL values between the MM-PBSA and MM-GBSA analyses revealed significant differences in the calculated binding free energies. For instance, in the V2-RP-105 complex, while both methods showed favorable binding (with ΔTOTAL values of -49.22 kcal/mol in MM-PBSA and -117.38 kcal/mol in MM-GBSA), the MM-GBSA yielded a significantly more negative value. Similarly, in the V2-TLR-9 complex, the MM-GBSA (-169.86 kcal/mol) predicted a much lower binding free energy than MM-PBSA (-0.17 kcal/mol). For V2-RP-105, the MM-PBSA and MM-GBSA revealed contrasting ΔEEL values (-504.90 kcal/mol in MM-PBSA and -984.03 kcal/mol in MM-GBSA), indicating significant sensitivity to the solvation model. Moreover, the ΔEEL calculated for the V2-TLR-9 using the MM-PBSA (-356.94 kcal/mol) significantly differed from that obtained with the MM-GBSA (-985.1 kcal/mol). However, both suggested notable electrostatic interactions between the vaccines and receptors. The ΔVDWAALS also showed a disparity between the MM-PBSA and MM-GBSA, where the MM-GBSA predicted a more favorable interaction (-244.0 kcal/mol for the V2-RP-105 and -228.68 kcal/mol for the V2-TLR-9) compared to the MM-PBSA (-153.47 kcal/mol for the V2-RP-105 and -0.28 kcal/mol for the V2-TLR-9). The ΔEGB and ΔESURF also exhibited significant differences between the MM-PBSA and MM-GBSA analyses. Regarding the V2-RP-105, the MM-GBSA predicted a higher ΔEGB (1142.64 kcal/mol and 629.01 kcal/mol) and ΔESURF (-31.95 kcal/mol and -19.86 kcal/mol) compared to the MM-PBSA. Similarly, in the V2-TLR-9, the ΔEGB (1074.7 kcal/mol and 357.06 kcal/mol) and ΔESURF (-30.79 kcal/mol and 0.00 kcal/mol) were predicted to be high in MM-GBSA compared to the MM-PBSA.

Using the NMA analysis, the stability and dynamic performances of the V2-apo, V2-RP-105, and V2-TLR-9 complexes were also assessed. The illustrations, thus, depicted the degree of stability of the applied complexes. Aiming for an enhanced expression of the recombinant vaccine (V2), codon optimization was performed using the *E*. *coli* expression system, explicitly using the K12 strain. The experiment results indicated that the V2 had a high degree of expression, as shown by its CAI value and average GC content of 0.9607 and 52.34%, respectively. Furthermore, it was shown that the V2 can elicit both innate and adaptive immune responses. The development of memory B-cells and T-cells was obtained, and the immunity of the B-cells lasted for a year. Noticeable features were seen in the activation of TH, the consequent production of IFN-γ and IL-2 after the first injection, and their continued elevation with successive antigen exposure. This finding suggests a humoral immune response since more TH cells and immunoglobulins (IgM and IgG) are being synthesized.

## 5. Conclusion

Using computational approaches has paved the way for developing a multiepitope vaccine against *Echinococcus* spp. The most promising epitopes derived from the Ag5 protein were chosen based on high antigenicity, conservancy, reduced allergenicity and toxicity. Molecular dynamic simulations and docking studies validated the vaccine’s binding affinity to the immune receptors, highlighting its potential efficacy upon administration. Furthermore, codon optimization confirmed the enhanced expression of the designed vaccine in *E*. *coli* systems. Notably, the vaccine demonstrated the ability to elicit robust innate and adaptive immune responses, with sustained humoral immunity observed over time. These findings underscore a promising prospect of the designed vaccine in combating echinococcosis and suggest avenues for further preclinical and clinical investigations. Also, the findings provide some novel and essential epitope choices and present an innovative approach for developing an echinococcosis vaccine, which is the very first attempt in Bangladesh, even in the world.

## Supporting information

S1 Table Secondary structure prediction by SOPMA and GOR4 server. (DOCX)

S2 Table ElliPro predicted the discontinuous B-cell epitope residues of the V2 structure. (DOCX)

S1 Fig The pairwise sequence alignment between the Ag5 proteins of *E*. *granulosus* and *E*. *multilocularis*. (TIF)

S2 Fig The secondary structures of the V1 (A & C) and V2 (B & D) with their detailed features and structures. (TIF)

S3 Fig The tertiary structures of the V1 (A & B) and V2 (C & D), showing surface and ribbon like structures. (TIF)

S4 Fig The superimposed structure of the V2, before (cyan color) and after (yellow color) refinement. (TIF)
