# Supplementary material for: Designing of a multiepitope-based vaccine against echinococcosis utilizing the potent Ag5 antigen: Immunoinformatics and simulation approaches
Source: PLoS One. 2025 Feb 12;20(2):e0310510. doi: 10.1371/journal.pone.0310510 (PMC11819600; doi:10.1371/journal.pone.0310510)
Supplement: S1 Table — (DOCX) [file pone.0310510.s001.docx]

| **Properties** | **SOPMA** | | **GOR4** | |  |
| --- | --- | --- | --- | --- | --- |
|  | **V1** | **V2** | **V1** | **V2** |  |
| Alpha helix (Hh) | 63 (19.44%) | 138 (35.38%) | 47 (14.51%) | 132 (33.85%) |  |
| 3_10_ helix (Gg) | 0.00% | 0.00% | 0.00% | 0.00% |  |
| Pi helix (Ii) | 0.00% | 0.00% | 0.00% | 0.00% |  |
| Beta bridge (Bb) | 0.00% | 0.00% | 0.00% | 0.00% |  |
| Extended strand (Ee) | 57 (17.59%) | 55 (14.10%) | 67 (20.68%) | 58 (14.87%) |  |
| Beta turn (Tt) | 33 (10.19%) | 44 (11.28%) | 0.00% | 0.00% |  |
| Bend region (Ss) | 0.00% | 0.00% | 0.00% | 0.00% |  |
| Random coil (Cc) | 171 (52.78%) | 153 (39.23%) | 210 (64.81%) | 200 (51.28%) |  |
| Ambiguous states (?) | 63 (19.44%) | 0.00% | 0.00% | 0.00% |  |
| Other states | 0.00% | 0.00% | 0.00% | 0.00% |  |

**S1 Table.** Secondary structure prediction by SOPMA and GOR4 server.

**S2 Table.** ElliPro predicted the discontinuous B-cell epitope residues of the V2 structure.

| **No.** | **Residues** | **Number of residues** | **Score** |
| --- | --- | --- | --- |
| 1 | A:M1, A:A2, A:K3, A:L4, A:S5, A:T6, A:D7, A:E8, A:L9, A:L10, A:D11, A:A12, A:F13, A:K14, A:E15, A:M16, A:T17, A:L18, A:L19, A:E20, A:L21, A:S22, A:D23, A:F24, A:V25, A:K26, A:K27, A:F28, A:E29, A:E30, A:T31, A:S32, A:E33 | 33 | 0.873 |
| 2 | A:V34, A:T35, A:A36, A:A37, A:A38, A:P39, A:V40, A:A41, A:V42, A:A43, A:A44, A:A45, A:G46, A:A47, A:A48, A:P49, A:A50, A:G51, A:A52, A:A53, A:V54, A:E55, A:A56, A:A57, A:E58, A:E59, A:Q60, A:S61, A:E62, A:F63, A:D64, A:V65, A:I66, A:L67, A:E68, A:A69, A:A70, A:G71, A:D72, A:K73, A:K74, A:I75, A:G76, A:V77, A:V85, A:S86, A:G87, A:L88 | 48 | 0.738 |
| 3 | A:Y165, A:F166, A:Q169, A:S171, A:H172, A:G173, A:G174, A:F175, A:Y176, A:F177, A:A178, A:K179, A:V180, A:D181, A:S182, A:P183, A:F184, A:D185, A:V186, A:A187, A:L188, A:L189, A:A190, A:K191, A:S193, A:L194, A:F195, A:V196, A:Y197, A:R198, A:E199, A:N200, A:I201, A:Y202, A:D203, A:G204, A:W205, A:S206, A:A207, A:Y208, A:Y209, A:L210, A:G211, A:L212, A:E213, A:L214, A:R238, A:N274, A:I275, A:Y276, A:G278, A:A279, A:Y280, A:Y281, A:K282, A:P283, A:I284, A:F285, A:G286, A:S287, A:S288, A:N289, A:A290, A:L291, A:P292, A:F293, A:G294, A:I295, A:P296, A:A297, A:P298, A:L299, A:N300, A:T301, A:D302, A:G311, A:E312, A:G313, A:T314, A:R315 | 80 | 0.619 |
| 4 | A:A100, A:P101, A:K102, A:P103, A:L104, A:L105, A:E106, A:K107, A:V108, A:A109, A:E137, A:Y138, A:E139, A:S140, A:R141, A:L142, A:P143, A:T362, A:S363, A:G364, A:P365, A:G366, A:P367, A:G368, A:K369, A:C370, A:F371, A:H372, A:H373, A:P379, A:G380, A:P381, A:G382, A:N383, A:K384, A:G385, A:V386, A:C387, A:A388, A:G389, A:D390 | 41 | 0.599 |
| 5 | A:D219, A:L221, A:V222, A:K223, A:A224, A:S241, A:P242, A:A243, A:Y244, A:F269, A:V270, A:Y271, A:E273 | 13 | 0.535 |
| 6 | A:Y144, A:A145, A:K146, A:I147, A:P148, A:E319, A:Q320, A:E321, A:S322, A:G323, A:P324, A:G325, A:P326, A:G327, A:K328, A:S329, A:R330, A:P331, A:I332, A:S333, A:K334, A:P335, A:R336, A:R337, A:P340, A:W348, A:P349 | 27 | 0.532 |
| 7 | A:D374, A:D375, A:E376, A:N377, A:G378 | 5 | 0.512 |
