# Supplementary figures and images for: Designing of a multiepitope-based vaccine against echinococcosis utilizing the potent Ag5 antigen: Immunoinformatics and simulation approaches
Source: PLoS One. 2025 Feb 12;20(2):e0310510. doi: 10.1371/journal.pone.0310510 (PMC11819600; doi:10.1371/journal.pone.0310510)

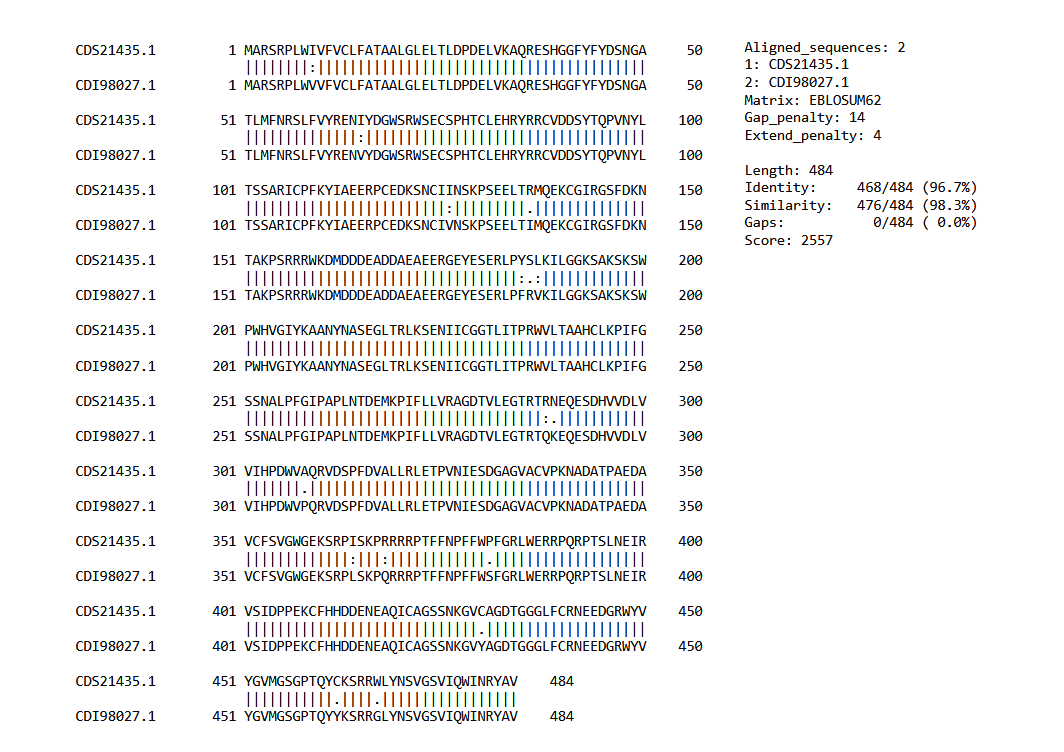

Supplement: S1 Fig — (TIF) [file pone.0310510.s003.tif]

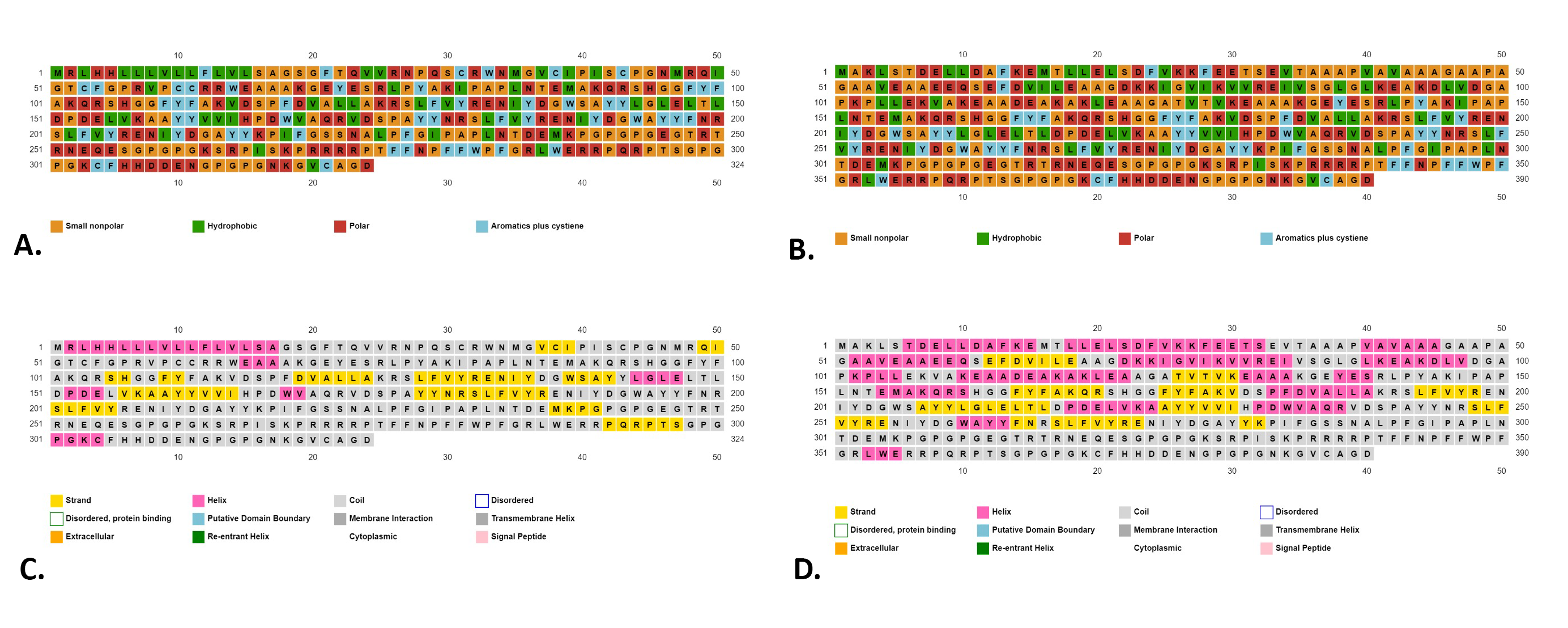

Supplement: S2 Fig — (TIF) [file pone.0310510.s004.tif]

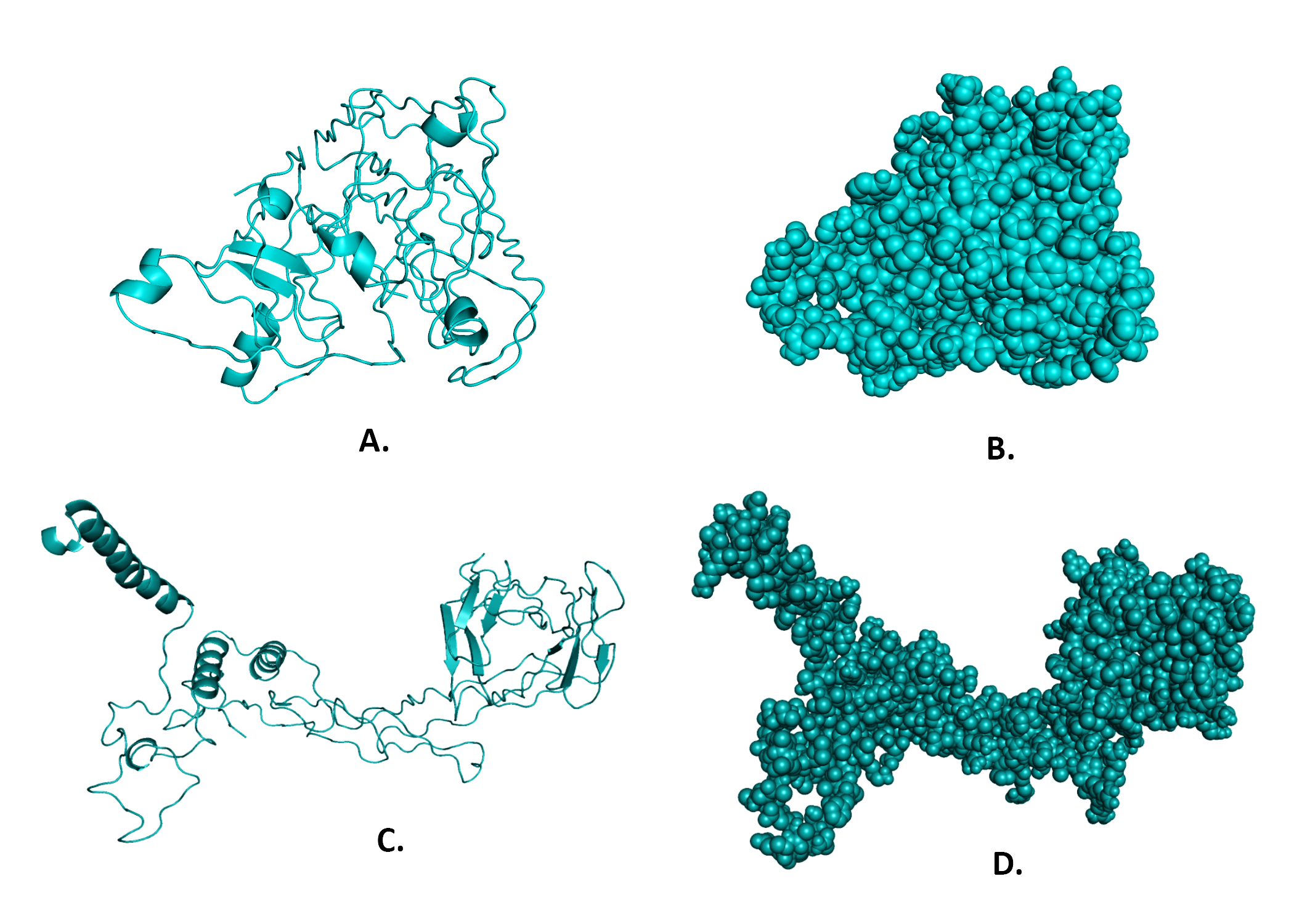

Supplement: S3 Fig — (TIF) [file pone.0310510.s005.tif]

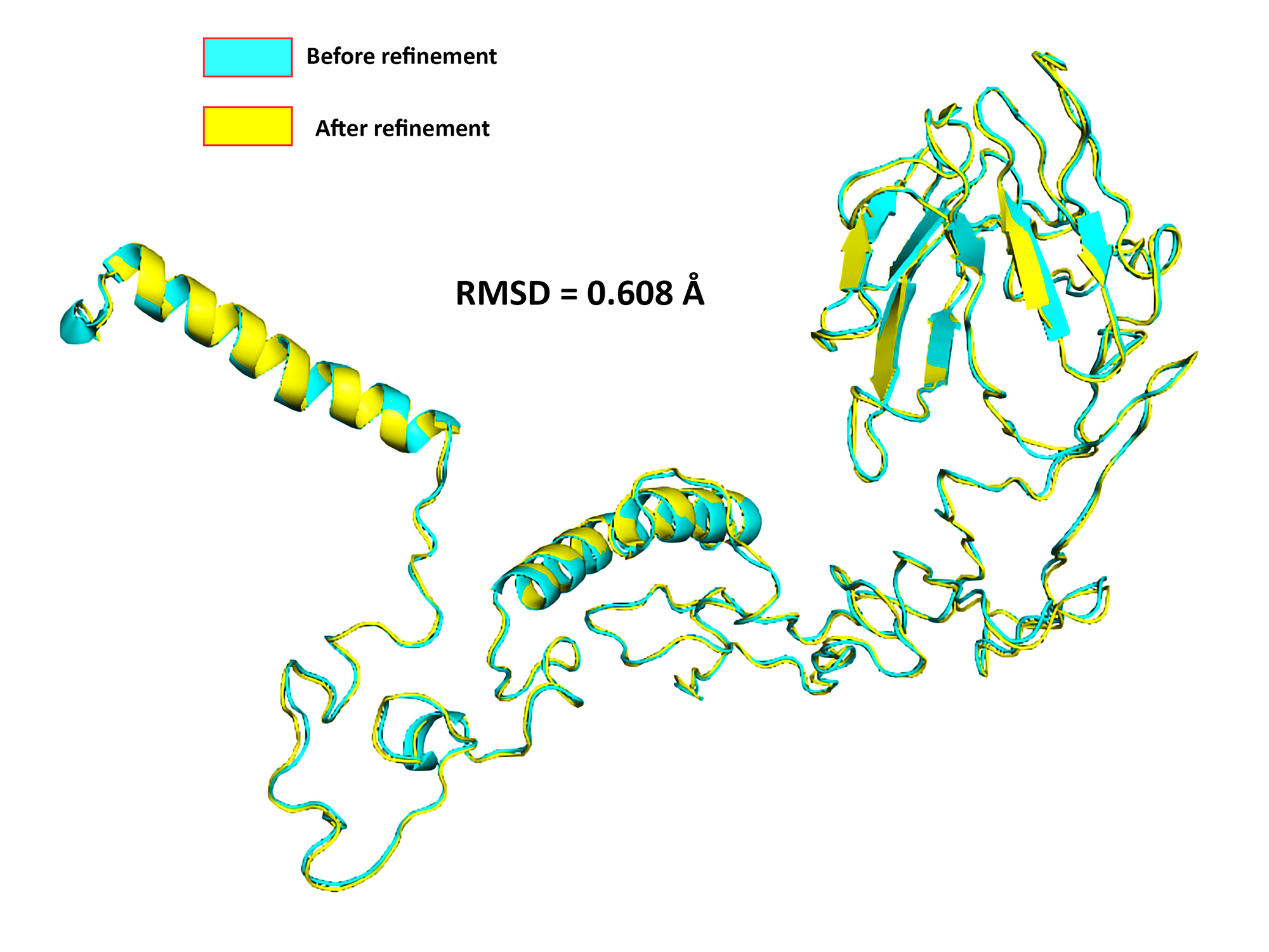

Supplement: S4 Fig — (TIF) [file pone.0310510.s006.tif]
